# Analysis of Rice Transcriptome Reveals the LncRNA/CircRNA Regulation in Tissue Development

**DOI:** 10.1186/s12284-021-00455-2

**Published:** 2021-01-28

**Authors:** Run Zhou, Pablo Sanz-Jimenez, Xi-Tong Zhu, Jia-Wu Feng, Lin Shao, Jia-Ming Song, Ling-Ling Chen

**Affiliations:** 1 National Key Laboratory of Crop Genetic Improvement, College of Informatics, Huazhong Agricultural University, Wuhan, 430070 People’s Republic of China; 2 College of Life Science and Technology, Guangxi University, Nanning, 530004 People’s Republic of China

**Keywords:** 3D genome, Epigenetic, Long non-coding RNAs (lncRNAs), Circular RNAs (circRNAs), Competing endogenous RNAs (ceRNAs), DNA methylation

## Abstract

**Background:**

Long non-coding RNAs (lncRNAs) and circular RNAs (circRNAs) can play important roles in many biological processes. However, no study of the influence of epigenetics factors or the 3D structure of the genome in their regulation is available in plants.

**Results:**

In the current analysis, we identified a total of 15,122 lncRNAs and 7902 circRNAs in three tissues (root, leaf and panicle) in the rice varieties Minghui 63, Zhenshan 97 and their hybrid Shanyou 63. More than 73% of these lncRNAs and parental genes of circRNAs (P-circRNAs) are shared among *Oryza sativa* with high expression specificity. We found that, compared with protein-coding genes, the loci of these lncRNAs have higher methylation levels and the loci of circRNAs tend to locate in the middle of genes with high CG and CHG methylation. Meanwhile, the activated lncRNAs and P-circRNAs are mainly transcribed from demethylated regions containing CHH methylation. In addition, ~ 53% lncRNAs and ~ 15% P-circRNAs are associated with transposable elements (TEs), especially miniature inverted-repeat transposable elements and RC/Helitron. We didn’t find correlation between the expression of lncRNAs and histone modifications; however, we found that the binding strength and interaction of RNAPII significantly affects lncRNA expression. Interestingly, P-circRNAs tend to combine active histone modifications. Finally, we found that lncRNAs and circRNAs acting as competing-endogenous RNAs have the potential to regulate the expression of genes, such as osa-156 l-5p (related to yield) and osa-miR444a-3p (related to N/P metabolism) confirmed through dual-luciferase reporter assays, with important roles in the growth and development of rice, laying a foundation for future rice breeding analyses.

**Conclusions:**

In conclusion, our study comprehensively analyzed the important regulatory roles of lncRNA/circRNA in the tissue development of *Indica* rice from multiple perspectives.

**Supplementary Information:**

The online version contains supplementary material available at 10.1186/s12284-021-00455-2.

## Background

Non-coding RNAs (ncRNAs), or RNAs which are not translated to proteins, are universally observed in eukaryotic genomes, and in plants ncRNA transcripts make up more than 50% of all RNA transcripts (Shin et al. 2018). Different functional ncRNAs have been identified with the rapid development of sequencing technology (Morris and Mattick 2014), including 18–24 nucleotide micro-RNAs (miRNAs) that participate in RNA silencing and post-transcriptional regulation (O'Brien et al. 2018), lncRNAs (> 200 bp) regulating gene expression and other processes (Cesana et al. 2011; Cheng and Lin 2013), and circRNAs that have covalently linked ends and are involved in transcriptional or post-transcriptional regulation (Chen et al. 2015). Competing endogenous RNAs (ceRNAs) are a class of coding genes/non-coding RNAs that share the same miRNA recognition elements (MREs), thereby competing for miRNA binding sites and regulating each other, adding complexity to the gene regulation network mediated by miRNAs (Wang et al. 2014; Liu et al. 2015). LncRNAs and circRNAs acting as potential ceRNAs can compete for the same MREs and therefore regulate protein expression (Memczak et al. 2013; Wang et al. 2013).

Asian rice (*Oryza sativa*), feeding approximately half of the human population, is one of the most important food crops worldwide and the best model for cereal genomics because its rich source of genetic diversity between species and its capacity to hybridize. Minghui 63 (MH63, *Oryza sativa xian/indica* II) and Zhenshan 97 (ZS97, *Oryza sativa xian/indica* I) are widely cultivated in China and southeast Asia, which are the parents of the elite rice hybrid Shanyou 63 (SY63), and important varieties in functional genomic studies (Zhang et al. 2016a; Wang et al. 2014). Besides, considering that lncRNAs and circRNAs are widely distributed in the genome, the three-dimensional (3D) configuration of the genome, which is complex, dynamic and crucial for gene regulation, may contain important information of lncRNAs and circRNAs. It is reported that 82% of the MH63 genome is in 3D chromatin interaction modules with different transcriptional activities (Zhao et al. 2019), making it very suitable for studying the 3D genomic characteristics of lncRNAs and circRNAs, which have not been explored up to now.

To uncover the comprehensive role that ncRNAs play in rice, we performed high-throughput sequencing analysis to identify and characterize lncRNAs and circRNAs from three different tissues in MH63, ZS97 and SY63, respectively. Then we characterized their genomic regions of origin, analyzed the influence of methylation, TEs and the chromatin 3D structure in their formation and between them, and uncovered their functions and interactions with miRNAs as ceRNAs in the regulation of gene expression. Our data provides an important resource for future ceRNA and 3D genome research and a genome-wide profiling of lncRNAs and circRNAs in rice, increasing our understanding of a crop that is essential for the quality, reliability and sustainability of the world’s food supply.

## Results

### LncRNAs and circRNAs Are Widely Distributed in Rice

**Fig. 1:**
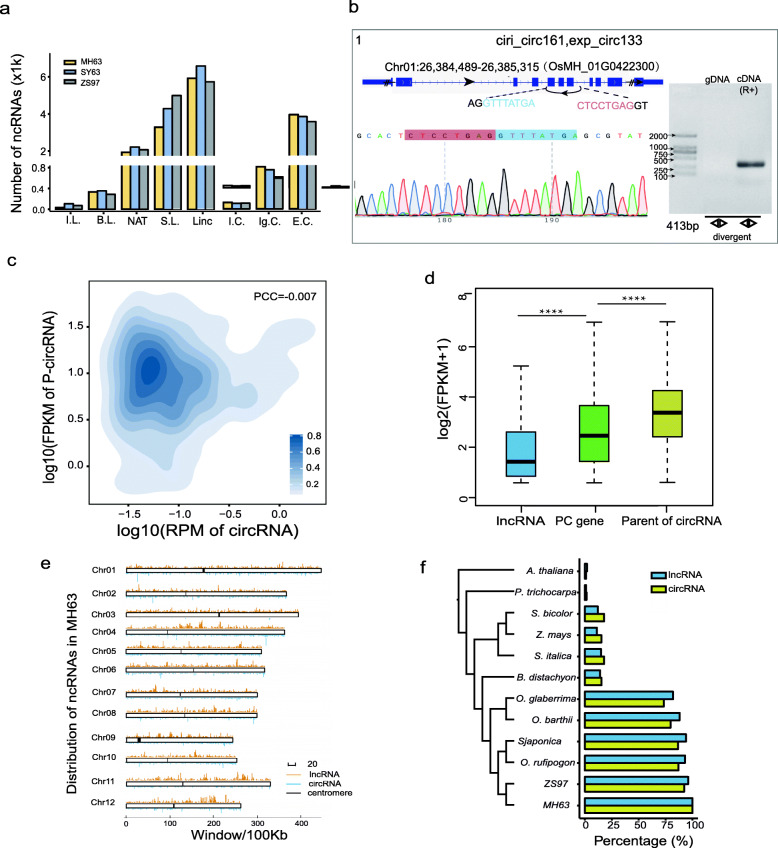
Characteristics and comparative analysis of lncRNAs, circRNAs and mRNAs in rice. **a** Classification of the lncRNAs and circRNAs in MH63, SY63 and ZS97. From left to right: Intronic lncRNAs, Bidirectional lncRNAs, lncNAT, Sense lncRNAs, lincRNA, Intronic circRNA, Intergenic circRNA and Exonic circRNA. **b** An example of CircRNA that was successfully amplified and sequenced for the validation. R+ represents sample with RNase R treatment. The black dotted line represents the splice site. **c** The relationship between the expression of circRNAs (RPM) and their parental genes (FPKM) in panicle of MH63. **d** Comparison of expression (FPKM) between lncRNA loci, PC genes and circRNA parental genes in MH63 (**** indicates *p*-value< 0.0001). **e** Distribution of the number of lncRNAs and circRNAs in the chromosomes of MH63. **f** The phylogenetic tree and conservation ratio of lncRNAs and circRNAs aligned to other plant genomes

About 1.3 billion read pairs were generated from 18 *Oryza sativa* L. ssp*. indica* samples, including three tissues (young leaf, panicle and root) from three rice varieties (MH63, ZS97 and SY63), with two replicates per tissue and variety (Table S1). Based on the pipeline in Fig. S1a, we identified 11,513, 13,153 and 13,549 lncRNAs in MH63, ZS97 and SY63, respectively (Fig. 1a, Table S2 and Additional table). LncRNAs were classified into five types, i.e., intergenic, intronic, antisense, bidirectional and sense lncRNAs, of which long intergenic non-coding RNAs (lincRNAs) and long non-coding natural antisense transcripts (lncNATs) accounted for the majority with 5723, 5923 and 6585 lincRNAs; and 1934, 2070 and 2216 lncNATs in MH63, ZS97 and SY63, respectively (Fig. 1a). LncRNAs that intersected with protein-coding (PC) genes in the sense strand (sense lncRNAs) possibly resulted from an incomplete annotation or a coding-free transcript of coding gene due to alternative splicing events. The Pearson correlation of lncRNA expression between two replicates per tissue was > 0.9, indicating the high repeatability of our data (Fig. S1b).

A total of 5122, 4273 and 4707 circRNAs (Additional table) were identified in MH63, ZS97 and SY63 after quality control and filtering. All samples showed similar values to their biological replicates (Table S3). Genome-wide annotation of these circRNAs included 4013, 3571 and 3847 exonic, 298, 112 and 112 intronic and 811, 590 and 754 intergenic circRNAs in MH63, ZS97 and SY63, respectively (Fig. 1a). Only ~ 25% of the circRNAs were shared by more than one tool in each variety (Table S4), which is similar to previous reports (Chen et al. 2018), highlighting the expected difference between different circRNAs tools.

CircRNAs identified with both CIRCexplorer2 (Zhang et al. 2016b) and CIRI2 (Gao et al. 2015) were considered as high-confident circRNAs. To further validate these circRNAs, RNA from young leaf at four-leaf stage of MH63 was extracted. We digested total RNA with RNaseR for 35 min and after running electrophoresis together with total RNA we confirmed the unambiguous presence of the main rRNA bands (18 s and 28 s) in total RNA, and their absence under RNaseR treatment (Fig. S2a). Then, we amplified leaf cDNA (RNaseR+), as well as genomic DNA (gDNA) using pairs of divergent primers of 30 high abundant exonic circRNAs, and of a non-circular (NC) RNA as control (Table S5). In total, 27 out of 30 circRNAs (90%) were successfully amplified with the expected length in cDNA (RNaseR+) while not in gDNA. As expected, linear RNA was amplified in gDNA while not in cDNA (RNaseR+). Then, Sanger sequencing showed that all 27 products amplified by divergent primers crossed the junction site of circRNAs (Fig. 1b, Fig. S2 and Table S5), confirming the reliability of our high-confidence circRNAs in this study.

First, we studied the genomic characteristics of these lncRNA and circRNA and found that the expression levels of circRNAs were not associated with their parental genes, as only 4% circRNAs were significantly positively correlated with the expression of their parental genes (*P*-value < 0.05, PCC > 0) (Fig. 1c). Furthermore, more than 30% of the parental genes produced more than one circRNA in each variety (Table S6), confirming that there is alternative splicing of circRNAs in rice. As expected, the middle length of lncRNAs in rice was longer than circRNAs, while was shorter than the average length of mRNAs (916 bp, 1339 bp and 1793 bp in circRNA, lncRNA and mRNA, respectively) (Fig. S1c). In addition, the expression of lncRNAs was lower than of PC genes, which was also lower than of P-circRNAs (Fig. 1d). Some circRNAs were very long (> 10 kb), which might be caused by the presence of repetitive DNA in the plant genome (Mehrotra and Goyal,^ 2014^). In total, ~ 4.8% (196) of circRNAs > 10 kb and located in intergenic regions was filtered from subsequent analyses. Sequence analysis revealed that single exonic lncRNAs or circRNAs were the most common (Fig. S1d), accounting for 41% of lncRNAs, which was significantly higher than in mRNAs (23%) and circRNAs (20%). LncRNAs/circRNAs were not equally distributed in each chromosome, and the proportion of lncRNAs/circRNAs per length or number of genes in the chromosomes changed between chromosomes and varieties (Table S7, S8). There were six lncRNA clusters distributed in chromosomes 4, 9, 10, 11 and 12 (Fig. 1e). Among them, one lncRNA cluster located in 20–20.5 Mb of chromosome 12 was conserved among the three rice varieties.

To assess the conservation of lncRNAs/circRNAs in rice and other plant genomes, these sequences were aligned to the genomes of some representative plant species using BLASTN. More than 82% of the lncRNAs sequences in MH63 were conserved in the genomes of different rice varieties (Fig. 1f, Table S9), including ZS97, *Oryza sativa subsp. geng/japonica*, African cultivated rice (*Oryza glaberrima*) and wild rice (*Oryza barthii*, *Oryza rufipogon*). Similarly, the conservation of circRNAs in rice was ~ 73%. In contrast, analysis of the genomes of different monocots including *Brachypodium distachyon*, *Setaria italica*, *Zea may* and *Sorghum bicolor* showed similar low conservation in both lncRNAs (< 11%) and circRNAs (< 15%), while in dicots such as *Arabidopsis thaliana* and *Populus trichocarpa* the conservation was very low (1–2%). This reflects that lncRNAs/circRNAs were not conserved among different plant species, but had certain intra-species conservation (Fig. 1f), indicating that lncRNAs/circRNAs can be considered as “young” genes as they evolved relatively recently.

Further analyses revealed that 47% and 58% of the lncRNAs sequences from MH63 could align to those of ZS97 and SY63, respectively. The conservation analysis of the back-splicing junctions of circRNAs in MH63 showed that ~ 39% circRNAs were conserved in ZS97, and almost 51% could align to SY63 (Table S9). In addition, 3445 (30%) lncRNAs and 1361 (32%) circRNAs of MH63 were conserved in ZS97 and SY63, indicating that many lncRNAs/circRNAs had species-specific expression.

### DNA Methylations of lncRNAs and circRNAs in Rice Genome

**Fig. 2:**
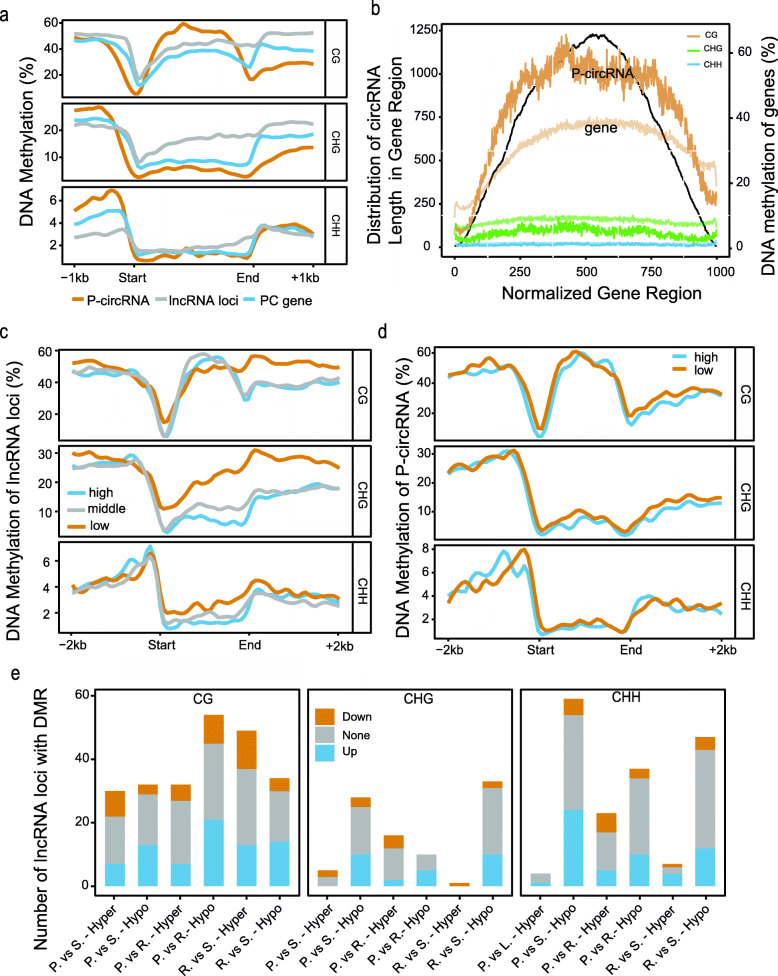
DNA methylation of lncRNA loci and P-circRNAs in MH63. **a** CG, CHG and CHH methylation densities in the parental genes of circRNAs (orange), lncRNAs locus (grey) and PC genes (cornflower blue) and their 1 kb up/down flanking regions. **b** Distribution of the positions of circRNAs (black) and DNA methylation (orange for CG, green for CHG and blue for CHH) in the P-circRNAs normalized into 1 kb length. DNA methylation levels of all PC genes were used as controls (light color represents PC genes). DNA methylation densities in the 2 kb up/down flanking regions of the TSS and TES of (**c**) lncRNAs and (**d**) circRNAs were categorized to low (first quantile, orange line) and high expression (three quantile, cornflower blue line). **e** Histogram showed the number of lncRNA loci containing hyper- and hypo-methylation on DMRs in body. LncRNAs were classified into groups as the same as a. DNA methylation (CG, CHG and CHH) profile of non-differently expressed (None, grey), upregulated (Up, orange), and downregulated (Down, cornflower blue) lncRNA loci in MH63. P. vs. S. referred to the comparison between panicle and seedling. P. vs. R. referred to the comparison between panicle and root. R. vs S. referred to the comparison between root and seedling

To further explore the regulation of lncRNAs/circRNAs in rice, we investigated DNA methylation, an important epigenetic modification in plants, which has been observed for CG, CHG and CHH contexts with H being any nucleotide but G. We analyzed the methylation densities of lncRNAs loci and the parental genes of circRNAs (P-circRNAs), and compared them with those of PC genes in MH63 genome. We found that lncRNAs had higher methylation level (CG = 38.0%, CHG = 15.4%, CHH = 2.2%) than PC genes (27.3%, 8.4%, 1.4%, wilcox.test, *P* < 2.2e-16 for CG, CHG and CHH, respectively), while the P-circRNAs had the higher CG and lower CHG than PC genes (36.0%, 4.7%, 1.1%, wilcox.test, P < 2.2e-16 for CG and CHG, Fig. 2a). DNA methylation is more likely to be associated with promoter regulatory regions (Zhong et al. 2013), therefore, we examined the average methylation signals within 1 kb upstream the transcriptional start site (TSS). The lncRNA loci displayed a relatively higher CG and lower CHH methylation densities than PC genes, while for P-circRNAs were the opposite. Although the CG methylation density was also reduced near the TSS of lncRNAs, it remained ~ 2-fold higher than that of the PC genes, and the CG methylation of P-circRNAs was approximately 2-fold lower than PC genes (Fig. 2a, Fig. S3a).

Using PC genes as control, we normalized P-circRNAs into 1 kb and compared the position distribution of circRNAs relative to their parental genes and DNA methylation density. We found that circRNAs were mainly concentrated in the middle of gene-body rather than two sides, and circRNAs were highly correlated with CG and CHG enrichment compared with PC genes (Fig. 2b and Fig. S3b). The lncRNAs were divided into three groups according to their expression (low, FPKM < 0.5; middle, 0.5 < FPKM < 2; high, FPKM> 2), and the average density of DNA methylation over the lncRNA loci were plotted. We observed that the DNA methylation level of lncRNAs with FPKM > 0.5 were similar, while lncRNAs with FPKM< 0.5 had different methylation level near TTS, indicating that lncRNA with FPKM< 0.5 might be incomplete (Fig. 2c), which can provide insights for screening high-confidence lncRNAs. Similarly, we analyzed the DNA methylation levels of P-circRNAs at different quantile expression levels and found that P-circRNAs with high expression levels showed lower methylation near TSS (Fig. 2d).

We further describe the impact of methylation changes on lncRNA loci and P-circRNAs based on the methylation data from (Zhao et al. 2020). In total, 889 and 1042 DMRs were located in the body of lncRNAs and P-circRNAs for different tissues, which involved 660 (8%) and 641 (22%) loci, respectively. However, about half of these DMR-containing genes were differentially expressed. Similar with previous report (Zhao et al., 2018), we observed that DMRs related with CHG and CHH were predominantly hypo-methylated, resulting in up-regulated expression of lncRNAs and P-circRNAs (Fig. 2e and Fig. S3c).

### LncRNAs and circRNAs Are Associated with Different TEs

**Fig. 3:**
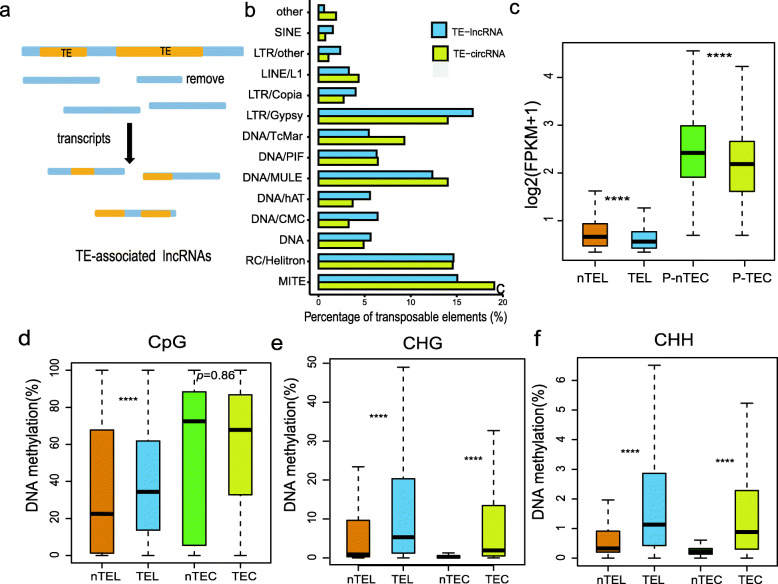
Transposable elements associated with lncRNAs and circRNAs in MH63. **a** Representation of TE-associated lncRNAs. **b** Distribution of TEs of the TE-associated lncRNAs and circRNAs. **c** Comparison of the expression (FPKM) in TE-associated (TEL) and non-TE-associated (nTEL) lncRNAs, and in the parental genes of TE-associated (P-TEC) and non-TE-associated (P-nTEC) circRNAs. Comparison of (**d**) CG, (**e**) CHG and (**f**) CHH DNA methylations between TE-associated and non-TE-associated lncRNAs (TEL, nTEL) and circRNAs (nTEC, TEC), respectively

Because DNA methylation in plant predominantly occurs on transposons (Law and Jacobsen 2010), after analyzing the methylation of lncRNAs and circRNAs we explored their relationship with transposable elements (TEs). First, we analyzed the influence of TEs on the origin of lncRNAs and circRNAs, and found that ~ 53% lncRNAs (4387) were associated with TEs (Fig. 3a), while only ~ 21.6% circRNAs (1106) were related to TEs, of which only 223 circRNAs were originated from TE-related genes in MH63. We observed that most (59% and 61%) TE-related lncRNAs/circRNAs are related to LTR/Gypsy, RC/Helitron, MITE and DNA/MULE-MuDR, both in quantity and length. In addition, we found the proportion of TE-related lncRNAs/circRNAs produced by RC/Helitron, MITE and DNA/MULE-MuDR elements to be relatively higher than their proportion in the genome (for example, RC/Helitron accounts for 11% in lncRNAs and 13% in circRNAs vs. 7% in genome). However, the contribution of LTR/Gypsy elements to TE-related lncRNAs/circRNAs was lower than its proportion in the annotated TEs (42%/34% vs. 46%). (Fig. 3b, Table S10). Through comparing the expression of TE-associated lncRNAs/circRNAs with non-TE-associated lncRNAs/circRNAs, we observed that the expression of non-TE-associated lncRNAs and P-circRNAs was higher than those associated with TEs (Wilcoxon’s test, *P* < 2.2e-16, Fig. 3c), while there was no difference in circRNA expression (Wilcoxon’s test, *P* > 0.05). We further analyzed the methylation profiles of TE-associated lncRNAs/circRNAs and found that all the methylation densities were significantly higher than those of non-TE lncRNAs/circRNAs (Fig. 3d-f), except that no difference was observed for CG methylation density between TE and non-TE-associated circRNAs. In short, the transposon elements, containing high DNA methylation levels, affected the expression of lncRNAs and P-circRNAs in *xian*/*indica* rice. In addition, the non-TE genes were more preferred to produce circRNAs than TE-related genes.

### RNAPII-Mediated Domains Affect Expression of lncRNAs in Rice

**Fig. 4:**
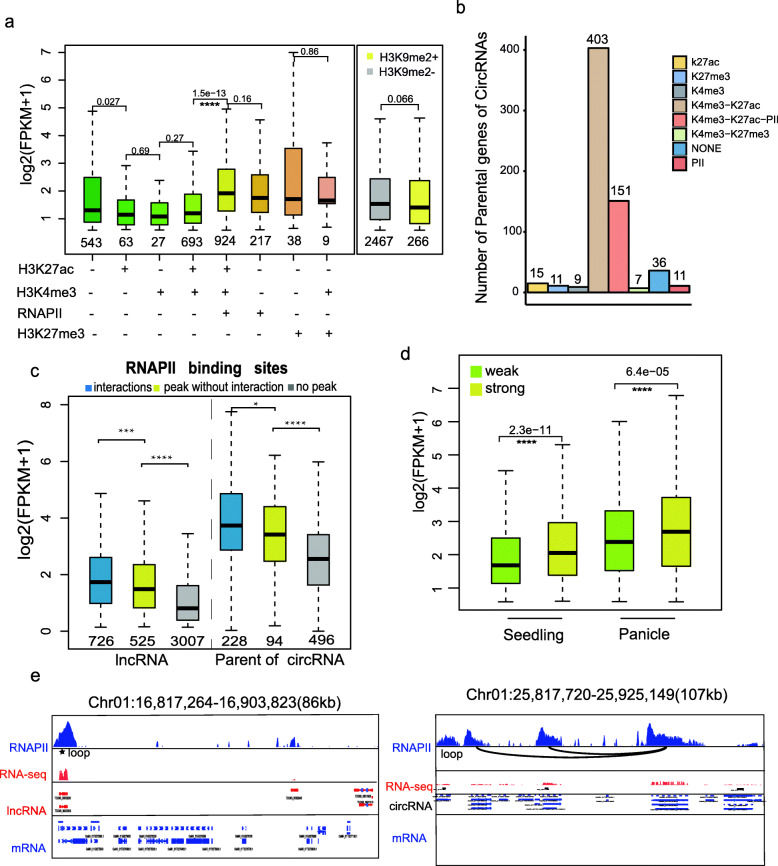
Comprehensive epigenome map of lncRNA and parental genes of circRNA in MH63 seedling. **a** The expression changes of lncRNA loci marked by different histone modifications and RNAPII. **b** Numbers of P-circRNA marked by different histone markers and RNAPII. (**c**) Comparison of the number and expression (FPKM) of lncRNAs and P-circRNAs in three types, i.e., those have interactions with RNAPII binding sites and have a peak, those have a peak but no interaction and no peak. **d** Expression levels of lncRNA loci marked by RNAPII with weak (Green) and strong intensity (yellow). **e** Example of one lncRNA (up) and circRNA (down) that have RNAPII binding site (blue), their expression (red) and interactions (loop)

To characterize the epigenomic feature and RNA polymerase II (RNAPII) occupancy in lncRNA and circRNA, we examined the expression of lncRNA loci and P-circRNAs harboring different histone marks. It was shown that the lncRNA loci marked by RNAPII were significantly highly expressed, however, lncRNAs marked by active histone (H3K4me3 and H3K27ac) or repressed histone (H3K27me3) had similar expression compared with those without histone markers (Fig. 4a, Fig. S4a). This phenomenon was different from protein-coding genes, which were activated by active histones including H3K27ac and H3K4me3 (Zhao et al. 2020). Therefore, P-circRNAs combined with active histone markers like H3K27ac and H3K4me3 explained the high expression of these genes (Fig. 4b, Fig. S4b and Fig. 1d). Although heterochromatin marker H3K9me2 accumulated mainly in the intergenic regions and exhibited significant higher levels of DNA methylation (Zhao et al. 2020), lncRNAs marked by H3K9me2 only accounted for 10% and had no significantly different expression than those not marked (Fig. 4a and Fig. S4c). These results suggested that histone modification may have distinct mechanism in lncRNAs compared with PC genes.

Previous reports highlight that lncRNAs and circRNAs have the potential to be transcribed by RNAPII (Sun et al. 2016; Wang and Chekanova 2017); however, a detailed functional mechanism for this process based on DNA 3D structure was lacking in plants. We analyzed the interaction characteristics of lncRNAs and circRNAs using RNAPII-mediated CHIA-PET database in MH63 (Zhao et al. 2019). It was observed that 29% lncRNAs (1251) and 39% circRNAs (322) were transcribed by RNAPII in seedling of MH63 (Fig. 4c), more than half of which were located in the RNAPII-mediated chromatin interaction domains (726 lncRNAs and 228 circRNAs, respectively). In order to determine if a quantitative relationship of intensity and expression was existed between RNAPII and lncRNA, we partitioned the RNAPII intensity in two groups (weak and strong), and plotted the expression of lncRNA loci contained in each group. We observed that lncRNAs harboring strong intensity of RNAPII were expressed higher than those with weak intensity of RNAPII (Fig. 4d, Fig. S4d). Notably, the expression of lncRNA loci and P-circRNAs in RNAPII-mediated interactions domains were much higher than those without interactions, and the lowest were those without RNAPII signals. For example, ciri_circ147, exp_circ113 and exp_circ114 contained RNAPII peaks with and without interaction but only had RPM value of 2.90e^− 08^, while exp_circ117 lacked RNAPII but had a higher RPM value of 2.61e^− 07^, further confirming that high expression of parental genes of circRNAs did not correlate with high expression of circRNAs (Fig. 4c, e).

### LncRNAs and circRNAs in Rice Show High Tissue Specificity

Both lncRNAs and circRNAs showed high tissue-specificity in all varieties, with panicle being the most abundant, then root and the last leaf in all the three rice varieties (Fig. S5a). In MH63, we identified 10,076 (88% account for total lncRNAs), 7517 (65%) and 6149 (52%) lncRNAs, and 3367 (66%), 1933 (38%) and 1160 (23%) circRNAs expressed in panicle, root and young leaf, respectively. Interestingly, tissue-specific expression of circRNAs was much higher than lncRNAs. We observed that ~ 43% lncRNAs were commonly expressed in three tissues and the rest were tissue-specific expressed, while the commonly expressed ratio of circRNAs was only 7.5%. In addition, we performed differential expression analysis for lncRNAs, and obtained 8419 (73%) differentially expressed lncRNAs (DELs) in MH63. GO analysis of the nearest genes of lncRNAs revealed that DELs were enriched in diverse biological processes, cellular components and molecular functions depending on the tissue of origin, confirming the dynamic expression of lncRNAs in different tissues. Nearest genes of up-regulated lncRNAs in panicle were enriched in ‘flower development and reproduction’, and in leaf were enriched in ‘transport and the formation of thylakoid and plastid’, and in root were enriched in ‘transcription factor activity’ (Fig. S5b). These enriched functions and the tendency of lncRNAs to cis-regulate the expression of nearby genes suggests that the DELs play an important role in the growth and development of different plant tissues. GO analysis for parental genes of circRNAs revealed that they can participate in ‘post-embryonic development’, ‘nitrogen compound metabolic process’, ‘binding of diverse molecules’ and ‘formation of different cell parts’ (Fig. S6a). Considering that circRNAs are highly dynamic and usually only expressed in one tissue, we also performed GO enrichment on parental genes of circRNA in each tissue, revealing that circRNAs participate in “reproductive and post-embryonic development’ in panicle, and contribute to ‘the formation of cytosol and cytoplasm’ in root (Fig. S5b). However, parental genes of circRNAs from leaf were not significantly enriched in any function when compared to those of panicle and root.

### LncRNAs and circRNAs Acting as ceRNAs Can Regulate Important Biological Traits in Rice

**Fig. 5:**
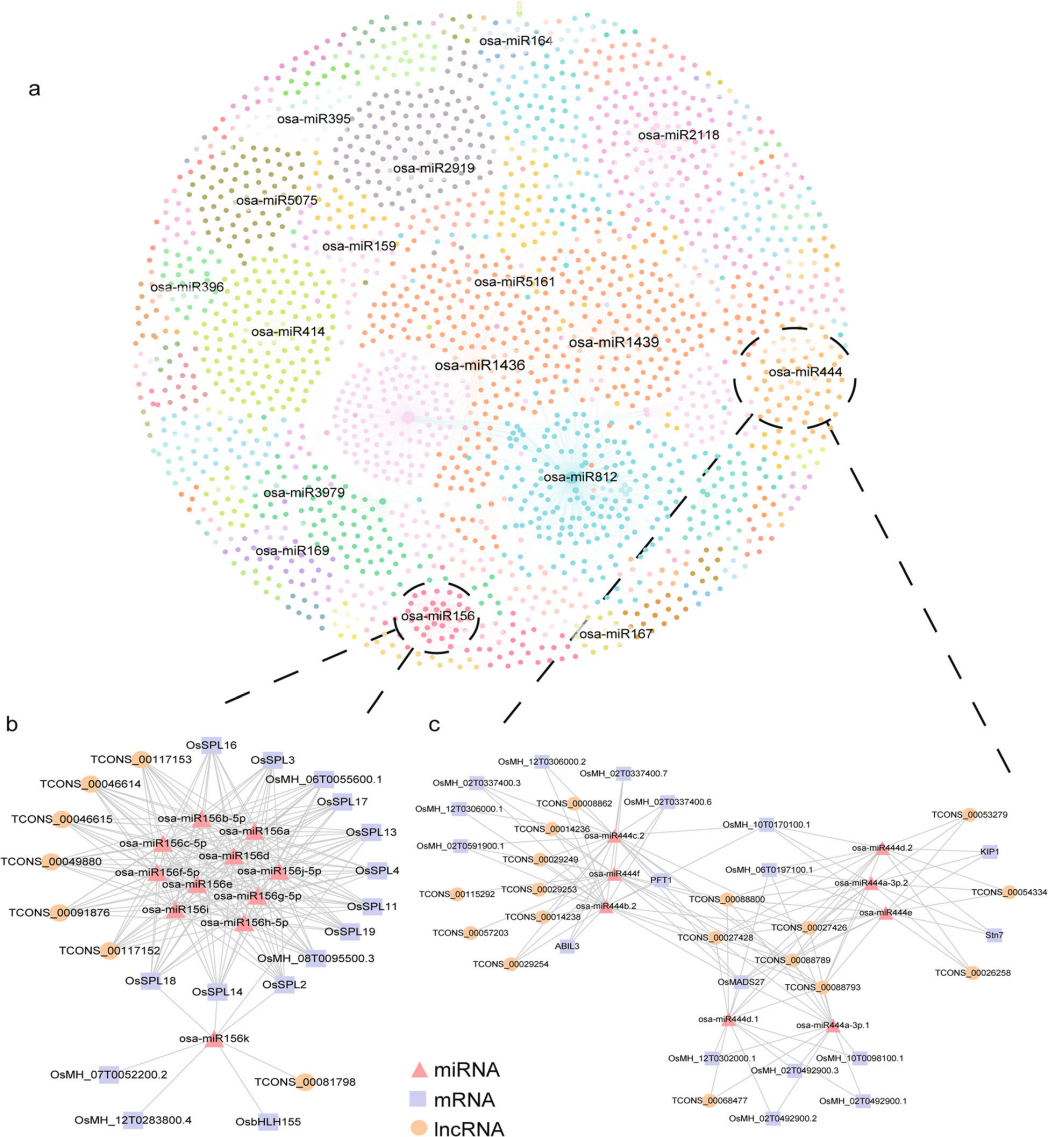
ceRNA network in MH63. **a** ceRNA network in which each color represents the different interactions of a miRNA. **b** osa-miR156 and (**c**) osa-miR444 miRNA families (red) ceRNA networks with lncRNAs (orange) and mRNAs (blue)

To evaluate the ncRNA-associated ceRNA interaction landscape in MH63, a complete circRNA/lncRNA-miRNA-mRNA interaction network was constructed with multi-tissues. In total, 797 lncRNAs and 215 circRNAs were interacted with 137 miRNAs and targeted a total of 1044 mRNAs, adding up to a total of 4517 different ceRNA pairs (Fig. 5a). Target genes of these miRNAs were enriched in flower development, reproduction, nucleus, nitrogen compound metabolic process and so on (Fig. S6b). Most of these miRNAs played significant roles in rice growth and development, important agronomic traits, and resistance to disease and drought. For example, Osa-miR156, which over-expressed in rice can increase salt stress tolerance and delay flowering (Wang et al., 2019). Another miRNA, Osa-miR444, is a key factor in relaying the antiviral signaling from virus infection and response to stress (Eren et al. 2015; Wang et al.^ 2016^). To understand the regulatory mechanisms of ceRNAs, we further explored the sub-networks related to the above two important miRNAs. We found that osa-miR156 can target and regulate OsSPL gene family members (Fig. 5b), including OsSPL14, a gene related to rice tillering, panicle number and thousand-grain weight (Miura et al.,^ 2010^). However, the mechanism of how osa-miR156 regulates OsSPL14 has not been reported. Our study suggests that lncRNA TCONS_00049880 located in intergenic region, may participate in the competitive binding of osa-miR156 and regulate the expression of SPL gene family. Similarly, several miRNAs from the osa-miR444 family in our network can target and regulate some transcription factors such as KIP1, PFT1, AP2/ERBP, MADS-box and calmodulin-binding protein (Eren et al. 2015). TCONS_00027428, a lncRNA located on the natural antisense strand of OsMADS27, can compete with OsMADS27 to combine osa-miR444a, a miRNA that plays multiple roles in the rice NO_3_^−^ signaling pathway in nitrate-dependent root growth, nitrate accumulation and phosphate-starvation responses (Yan et al. 2014).

To further confirm that lncRNAs TCONS_00049880 and TCONS_00027428 target miR156l-5p and osa-miR4443a-3p, respectively, we cloned the fragment of the lncRNAs containing the miRNA target sequence and established a luciferase construct. Transfection of miRNA suppressed significantly the luciferase activity of lncRNA in 293 T cells (Fig. S7), indicating that TCONS_00049880 competes with SPL family to bind osa-miR156l-5p and, similarly, that TCONS_00027428 competes with OsMADS27 to bind osa-miR444a-3p.

Finally, to explore the difference of ceRNA networks among different tissues, we further constructed circRNAs/DELs-miRNA-DEMs networks in MH63 for panicle, leaf and root. In total, 61, 40 and 52 miRNAs were predicted as target of 231, 68 and 101 DELs and 64, 72 and 99 circRNAs, and could interact with 180, 162 and 225 DEMs in panicle, leaf and root, respectively (Fig. S8, S9, S10). Difference in the ceRNA networks between tissues indicated that different lncRNAs and circRNAs participated in diverse functions in the growth and development of each tissue. We performed GO analysis of the target-genes and found that they were enriched in ‘flower development and reproduction’ in panicle, indicating a specific function of the DELs and circRNAs with miRNA recognition elements in panicle in regulating gene expression (Fig. S6b).

## Discussion

The plant epigenome research falls behind that of mammalians, not to mention epigenetic of non-coding RNA. In this study, we produced high coverage whole transcriptome sequencing and combined with publicly available dataset, including ChIP-Seq, CHIA-PAT, whole-genome bisulfite sequencing, *etc*, to reveal comprehensive epigenetic characteristics of lncRNA in rice. Based on these datasets with multiple tissues in three varieties, a high-resolution 3D architecture and epigenetic landscape of rice genomes were constructed, nearly 82% of the rice genomes was annotated, including DNA methylation, active or repressive DNA regulatory elements, euchromatin and heterochromatin (Zhao et al. 2019; Zhao et al. 2020). Considering that most studies only focus on protein coding genes, a possible link of 3D architecture and epigenome with lncRNAs and circRNAs in plants was not explored up to now.

We investigated the epigenetics involving expression of lncRNAs is depended on changes in gene dosage (e.g., copy-number alterations) and promoter utilization (e.g., DNA methylation) and their regulation since there are reports linking expression of circRNAs with DNA methylation in humans (Ferreira et al. 2018). Similar with previous research, DNA methylation of promoter was negatively correlated with gene expression levels in lncRNAs and P-circRNAs, which was also the same with protein-coding genes. DNA methylation density was significantly decreased near the transcription initiation site, especially in P-circRNA. Interestingly, we found a strong correlation between CG methylation and the distribution of circRNAs in the parental genes. In addition, DNA methylation levels of low-expressed (FPKM< 0.5) lncRNAs were significantly different near TTS compared with highly expressed lncRNAs, indicating that this part of lncRNAs may be incomplete, which provides a screening threshold for high-confidence lncRNAs. Recent study on the epigenetic landscape of cancer cells (Wang et al. 2018) and polyploid cotton (Zhao et al. 2018) showed that the lncRNA loci were hypo-methylated which was similar in this study and accompanied with the up-regulated of lncRNA loci, indicating that DNA methylation-mediated lncRNA regulation is a common mechanism in plants and animals.

TEs are normally transcriptionally silent regions due to DNA methylation. In rice, siRNAs have been associated with MITEs produced by OsDCL3a, which mediate DNA methylation (Wei et al. 2014), and TEs have been associated with variation of the expression of circRNAs (Lu et al., 2015). In our results, more than half (53%) of lncRNAs were associated with TE, and only a small amount (15%) of circRNAs were derived from TE-related genes. LncRNAs and circRNAs showed a clear consistent association with TEs with a high proportion of MITE and RC/Helitron in rice. The parental genes of circRNAs and lncRNAs that associated with TE correlated with low expression and high methylation, except for CG in circRNAs.

Furthermore, we analyzed the epigenetic profile of lncRNAs and found that more than half lncRNAs (56% in panicle, 71% in root, 72% in seedling) contains a variety of histone modifications, including active histone modifications (H3K4me3 and H3K27ac) and suppressed histone modifications (H3K27me3 and H3K9me2). Unlike active histone modification activating gene expression and suppressive modification silencing gene expression, histone modification near lncRNA had little influence on its expression. However, further analysis indicated that the nearest genes of lncRNAs combined with active histones (H3K4me3, H3K27ac) had significant higher expression than those without histone modification; while the characteristics of the nearest genes of lncRNAs combined with repress histone H3K37me3 had the opposite trend (Fig. S4e), suggesting that lncRNAs involving epigenetic modifications to specific genomic loci, which then regulated the expression of neighboring protein-coding genes (Wang et al. 2018). Hundreds of lncRNAs have been confirmed to interact with multiple proteins, for example, a lncRNA-LAIR significantly binding with RNA-binding proteins OsMOF and OsWDR5, which have shown to associate with the H3K4me3 and H4K16ac histone modification complex, suggesting lncRNAs influence gene expression by targeting chromatin remodelers to specific genomic regions as part of a molecular scaffold (Trapnell et al. 2012; Wang et al. 2018). Not surprisingly, the parental genes of circRNA were enriched with a large of active histone modifications. Similar with previous reports (Kindgren et al. 2018), many (~ 29% in seedling) lncRNAs were transcribed by RNAPII. In addition, our research revealed that the high activity and interaction of RNAPII promoted lncRNA transcription.

Finally, ceRNAs interactions have opened a new way of understanding the cross-regulation of mRNAs among different ncRNAs. A ceRNA interaction involving lncRNAs and circRNAs in *A. thaliana* has been reported (Meng et al. 2018), finding a correlation between ceRNAs and seedling development. Our ceRNA network revealed that less than 10% of the lncRNAs and circRNAs had miRNA interaction domains, meaning that in rice the “miRNA sponge” function is limited to a few lncRNAs and circRNAs. The lncRNAs/circRNAs acting as ceRNAs in our network have the potential to regulate the expression of genes with important functions like flower development and reproduction through binding to miRNAs. Due to the dynamic character of ncRNAs, we further constructed three tissue specific ceRNA networks with the circRNAs and up-regulated lncRNAs in panicle, seedling and root and, as we anticipated, the functions of the genes that they could regulate when interacting with miRNAs are different in each tissue, revealing in plants the tissue-specificity of the ceRNA interactions. Our dual-luciferase report experiment verified that lncRNAs can target osa-156 l-5p and osa-miR444a-3p to regulate the expression of related functional genes, verifying the reliability of our ceRNA networks and opening the way for future rice breeding analyses.

## Conclusions

Taken together, our study is one of the most complete analyses of ncRNAs, their genomic regions and the different factors that affect the regulation of their expression in rice, revealing the functions of these ncRNAs and the genes that they modulate through competing interaction mechanisms. The correlation between CG methylation and genomic region of the circRNAs, the inverse correlation between methylation in the gene body and expression, the different TEs and interaction domains preferences and the limited function as “miRNA sponge” of circRNAs and lncRNAs bring useful information for understanding ncRNAs in plants.

## Materials and Methods

### Plant Materials, RNA Library Construction and Sequencing

*Oryza sativa ssp. xian/indica* varieties MH63, ZS97 and SY63 were grown in a greenhouse of Huazhong Agricultural University, Wuhan (China), at 25 °C in non-stress conditions. Leaf and root samples at the seedling stage of 4 leaves and IV ~ V stage of panicle were collected for RNA isolation. A total amount of 3 μg was used as input for RNA sample preparation. Ribosomal RNA was removed by Epicentric Ribo-zero rRNA Removal Kit (Epicentre, USA), and rRNA free residue was cleaned up with ethanol precipitation. rRNA-depleted RNA was used for library construction according to NEBNext Ultra Directional RNA Library Prep Kit for Illumina (NEB, USA). In order to select cDNA fragments of preferentially 250–300 bp, the library fragments where purified with AMPure XP system (Beckman Coulter, Beverly, USA). Then 3 μL of USER Enzyme (NEB, USA) was used with size-selected, adaptor-ligated cDNA at 37 °C for 15 min followed by 5 min at 95 °C before PCR primers and Index (X) Primer. At last, products were purified (AMPure XP system) and library quality was assessed on the Agilent Bioanalyzer 2100 system. The clustering of the index-coded samples was performed on a cBot Cluster Generation System according to the manufacturer’s protocol. After cluster generation, the library preparations were sequenced on an Illumina Hiseq3000 that generated pair-end reads of 150 bp in length. Finally, cDNA primer and adaptor were filtered out and 10 bp from the start were trimmed out with Trimmomatic v0.32 (Bolger et al. 2014) after and before quality control analyses of the reads with FastQC to obtain clean reads. The data is shown in Table S1.

### Bioinformatics Pipeline for Characterization of lncRNAs

Clean reads were aligned to the MH63RS2 and ZS97RS2 reference genomes using Hisat2 v2.0.4 (Kim et al. 2015) and assembled with Cufflinks v2.2.1 (Trapnell et al. 2012). First, the individual samples were assembled separately, merged together and the expression level of each transcript was normalized using FPKM value. Next, the transcripts shorter than 200 bp, transcripts with mean FPKM scores < 0.1 and known protein-coding transcripts were filtered. The filtered sequences were used for hmmscan search against PFam-A 31.0 (El-Gebali et al. 2019). The transcripts which matched to protein family domain with e-value > 10^− 5^ were removed. The remaining transcripts were subjected to coding potential calculation using PlncPro (Singh et al. 2017) with monocot model (Coding Probability < 0.5). The remained transcripts were considered as reliably putative lncRNAs. Finally, lncRNAs were classified into intergenic, intronic, antisense, bidirectional and sense lncRNAs based on the known positions of adjacent protein-coding (PC) genes using FEElnc v.0.1.1 classifier (Wucher et al. 2017). In addition, the distribution of lncRNAs on the genome with 100 kb per bin was analyzed. If a region contained the number of lncRNAs > 2 of the average in each bin and the total length larger than 500 kb, it was considered as a hot lncRNA cluster.

### Computational Identification, Filtering and Characterization of Circular RNAs

Due to the reported difference in circRNA identification among different tools (Hansen et al. 2016), several widely used bioinformatics tools were employed for identifying circRNAs, including CIRI2 v2.0.6 (Gao et al. 2015), CIRCexplorer2 v2.3.5 (Zhang et al. 2016b) and find_circ v1.2 (Memczak et al. 2013). To obtain the most reliable results with each bioinformatics tool, we followed their recommended protocols for the identification and filtering of candidate circRNAs. First, sequencing reads were aligned to MH63RS2 and ZS97RS2 genomes  (Song et al, 2018). Then a series of different quality control steps were implemented for the inclusion of only high confident circRNAs for further analyses. For the candidate circRNAs detected with find_circ, the recommended filtering criteria for the software were applied. We required at least two reads supporting the candidate circRNA junction, unambiguous detection of the breakpoint, unique anchor alignments on both sides of the junction, and removed splice sites that were more than 100 kb. In addition, due to the reported lower accuracy of find_circ (Hansen et al. 2016), we further filtered the candidates with an in-house perl script, and those classified as exonic-, intronic- or intergenic- circRNA were kept (Memczak et al. 2013). Meanwhile, at least two junction reads covering the back splicing sites of the candidate circRNAs detected by CIRI2 and CIRCexplorer2 were required.

First the individual samples were assembled separately, and then merged together for each variety. The genomic region of the circRNAs in the parental genes was considered as the genomic region between both back-splicing junctions that corresponded with the circRNA strand. Gene expression levels were obtained with Cufflinks v2.2.1 (Trapnell et al. 2012) and normalized as Fragments Per Kilobase of transcript per Million mapped reads (FPKM). Expression levels for circRNAs were normalized as Reads Per Million mapped reads (RPMs).

### Experimental Validation of circRNA

To validate the reliability of the circRNAs, young leaf at four-leaf stage of MH63 were extracted under the same conditions as other samples of this study. The PCR primers were designed for the validation of 30 highly abundant circRNAs identified both in CIRI2 v2.0.6 and CIRCexplorer2 (Table S5). First, gDNA (FastPure Plant DNA Isolation Mini Kit) and total RNA (QIAsymphony RNA Kit) were extracted, and then 1ul of RNaseR was used to digest 5μg of total RNA. After a digestion time of 35 min to obtain cDNA (RnaseR+), cDNA (RNase R+) and genomic DNA were used as templates for the validation of circRNAs by quantitative PCRs. The reagent of 2 × Taq Plus Master Mix II (Dye Plus, VAZYME, P212) was used for cDNA and gDNA amplification with the touchdown PCR program to detect the candidate circRNA templates (Reaction conditions of touchdown PCR program: 95 °C 3 min; 95 °C 20 s, 54 °C ~ 48 °C (touch down − 1 °C) 20 s, 72 °C 30 s, 6 cycles; 95 °C 20 s, 54 °C 20 s, 72 °C 30 s, 30 cycles; 72 °C 5 min, 12 °C 1 min). Finally, Sanger sequencings were performed on all PCR products.

### Conservation of lncRNAs and circRNAs

The lncRNAs and circRNAs identified in MH63 were used as query sequences, and Blastn v2.2.30 (Altschul et al. 1990) was used to align them to the lncRNA, circRNA back-splicing sequences and genomes of other plant species. The genome sequences of 10 plant species were downloaded from Ensembl Plants (Ensembl Plants) and compared with lncRNAs/circRNAs sequences. Similar to a previously reported strategy (Xu et al. 2018), the thresholds for screening *Oryza* genus were identity ≥80% and e-value < 10^− 5^, and for monocotyledonous and dicotyledonous plants, the cutoff query was identity ≥50% and e-value < 10^− 5^. To compare the conservation between lncRNAs sequences, we compared the sequences of lncRNAs to those of ZS97 and SY63 with the same threshold as for *Oryza* genus. To ensure reliable alignment results comparing back-splicing sequences from MH63 and those of ZS97 and SY63, we required the identity ≥95%, e-value < 10^− 5^ and a maximum of 1 gap.

### Methylation and TE Analysis of lncRNAs and circRNAs

In order to detect 5-methylcitosine modifications, bisulfite sequencing (BS-Seq) was performed to detect DNA methylation in the three rice varieties. Total genomic DNA was extracted from 12-days seedling leaves following the DNeasy plant mini kit (Qiagen) protocol. After library preparation and sequencing, Trim_galore v0.4.5 (TrimGalore webpage) was used as a quality control and to trim low-quality bases. Finally, Bismark v0.19.1 (Bismark webpage) was used for mapping the clean reads to rice genomes, allowing one mismatch in 20-nucleotide seed sequence (−N 1 -L 20). The reads not uniquely mapped were filtered. Bismark was then used to determine the methylation level at each cytosine using its methylation extractor command. Because there is no methylation in the chloroplasts of plant genome (Du et al. 2017), all methylation levels detected in the chloroplast genome were accounted as false discovery rate with the error rate of ~ 0.3% (Fojtova et al. 2001). To identify differentially methylated regions (DMRs), we downloaded the DMR database form Zhao et al. (Zhao et al. 2020) and conducted a binomial test (*P* ≤ 0.05) for each cytosine site and the difference in DNA methylation between two samples was 0.6 or above. Similar to other studies (Xu et al. 2018), we define TE-associated lncRNAs as those lncRNAs transcripts that include a TE-site within their boundaries, but are not fully included inside a TE site (Fig. 3a). Meanwhile, if the parent genes are annotated as TE-genes, the related circRNAs are defined as TE-associated circRNAs.

### Phylogenetic Tree Analysis

Protein sequences of PC genes in ten plant species were downloaded from Ensembl Plants (Ensembl Plants). The longest protein sequence for each gene was employed to search for homologous families using OrthoMCLv2.0 (−I 1.5) (Li et al. 2003). Next, MAFFT (Katoh et al. 2002) was applied to multiple-sequencing alignments on single copy homologous protein sequences and Gblocks (Castresana 2000) was used to extract the conserved sites. Phylogenetic tree was constructed using RAxML v8 (Stamatakis 2014) and visualized with FigTree (FigTree webpage).

### The Epigenome Feature Analysis of lncRNAs and circRNAs

To analyze the 3D epigenome genome structure of lncRNAs/circRNAs, we downloaded distribution of different histones (H3K4me3, H3K27ac, H3K27me3, and H3K9me2) and RNAPII with distinct modification potentiality (Zhao et al. 2019; Zhao et al. 2020), and mapped the lncRNAs/circRNAs on histones region. If 1-bp overlap existed between a histone and a gene or locus, the histone binding was thought to influence the expression of this locus. Considering the strong tissue-specificity of lncRNAs/circRNAs, and because the CHIA-PET data of RNAPII interaction were derived from young leaf, we only analyzed the lncRNAs and circRNAs from young leaf.

### Differential Expression and GO Enrichment Analysis

DESeq2 (Love et al. 2014) was applied to filter the differentially expressed mRNAs (DEMs) and differentially expressed lncRNAs (DELs). Fold change (FC) and false discovery rate (FDR) were used to filter differentially expressed genes under the following criteria: (a) FC > 2 or < 0.5; (b) FDR < 0.05. To analyze the function of circRNAs we extracted their parental genes, and for DELs their nearest adjacent PC gene. For the target gene sets, AgriGO online tool (AgriGO webpage) was used for enrichment analysis (FDR < 0.05), and we set *P*-value < 0.05.

### Construction and Analysis of the lncRNA/circRNA -miRNA-mRNA ceRNA Networks

To obtain high-quality lncRNAs/circRNAs acting as miRNA targets, miRNA-mRNA and miRNA-lncRNAs/circRNAs interactions were predicted with Targetfinder v1.7 (Whalen et al. 2016) and PsRNATarget (Dai et al. 2018) using default parameters, and filtered lncRNAs/mRNAs with FPKM < 0.5. Only experimentally verified miRNAs were downloaded from PMRD (Zhang et al. 2010). Then, expression correlations between lncRNAs/circRNAs and mRNAs were calculated using Pearson correlation coefficient (PCC). The lncRNAs/circRNAs-mRNA pairs with PCC > 0.8 and *P* < 0.05 were selected as co-expression pairs. For a given co-expression pair, both mRNA and lncRNAs/circRNAs in this pair were targeted with a common miRNA, and this miRNA-mRNA-lncRNAs/circRNAs was identified as co-expression competing triplet. The networks were visualized with Gephi v0.9.2.

### Luciferase Reporter Assay

We cloned the fragment of lncRNAs containing miRNA target sequences and established a luciferase construct as described in (Lv et al., 2020). The sequence of lncRNA were cloned into the pmirGLO Dual-Luciferase ExpressionVector (Promega, USA) to construct the corresponding reporter vectors. Those reporter vectors were co-transfected with mimics or NC into 293 T cells using Lipofectamine 2000. The luciferase assay was performed using the Dual-Luciferase Reporter Assay System (Promega, USA) and the enzymatic activity of luciferase measured using a PerkinElmer 2030 Multilabel Reader (PerkinElmer, USA).

## Supplementary Information

**Additional file 1.**

**Additional file 2: Figure S1.** Workflow and characteristics of lncRNAs and circRNAs in rice. **Figure S2.** qPCR of 27 circRNAs from young leaf of MH63. **Figure S3.** DNA methylation of P-circRNAs and lncRNA loci in ZS97. **Figure S4.** Comprehensive epigenome map of lncRNA and P-circRNA in different varieties. **Figure S5.** Tissue specificity of lncRNAs and circRNAs in MH63. **Figure S6.** Gene ontology analysis of the target genes in MH63. **Figure S7.** Luciferase reporter assays of lncRNAs in 297 T cells. **Figure S8.** CeRNA network of DECs and DELs of panicle in MH63. **Figure S9.** CeRNA network of DECs and DELs of seedling in MH63. **Figure S10.** CeRNA network of DECs and DELs of root in MH63.

**Additional file 3: Table S1.** Data of RNA-seq in this study. **Table S2.** Tissue distribution of ncRNAs. **Table S3.** Replicate distribution of circRNAs. **Table S4.** Software distribution of high-confidence circRNAs. **Table S5.** Primers list of 27 validated circRNAs. **Table S6.** Alternative splicing of circRNAs. **Table S7.** Genomic distribution of circRNAs. **Table S8.** Genomic distribution of lncRNAs. **Table S9.** Conservation of lncRNAs and circRNAs in MH63 with other species. **Table S10.** The proportion of number/length attributable to different TE superfamilies of TE-related lncRNAs/circRNAs.

## Data Availability

All the RNA-seq data supporting the results of this article have been deposited at National Genomics Data Center under accession No. CRA002886 (National Genomics Data Center).

## Abbreviations

3D: Three-dimensional
CeRNAs: Competing endogenous RNAs
CircRNAs: Circular RNAs
DEMs: Differentially expressed mRNAs
DELs: Differentially expressed lncRNAs
DMRs: Differentially methylated regions
FC: Fold change
FDR: False discovery rate
FPKM: Fragments per kilobase of transcript per million mapped reads
LncRNAs: Long non-coding RNAs
MH63: Minghui 63
MiRNAs: micro-RNAs
MITEs: Miniature inverted-repeat transposable elements
NcRNAs: Non-coding RNAs
P-circRNAs: Parental gene of circular RNAs
PC: Protein-coding
RNAPII: RNA polymerase II
RPMs: Reads Per Million mapped reads
TSS: Transcriptional start site
SY63: Shanyou 63
ZS97: Zhenshan 97

## References

[CR1] Altschul SF, Gish W, Miller W, Myers EW, Lipman DJ (1990). Basic local alignment search tool. J Mol Biol.

[CR2] Bolger AM, Lohse M, Usadel B (2014). Trimmomatic: a flexible trimmer for Illumina sequence data. Bioinformatics.

[CR3] Castresana J (2000). Selection of conserved blocks from multiple alignments for their use in phylogenetic analysis. Mol Biol Evol.

[CR4] Cesana M, Cacchiarelli D, Legnini I, Santini T, Sthandier O, Chinappi M, Tramontano A, Bozzoni I (2011). A long noncoding RNA controls muscle differentiation by functioning as a competing endogenous RNA. Cell.

[CR5] Chen I, Chen CY, Chuang TJ (2015). Biogenesis, identification, and function of exonic circular RNAs. Wiley Interdiscip Rev RNA.

[CR6] Chen L, Zhang P, Fan Y, Lu Q, Li Q, Yan J, Muehlbauer GJ, Schnable PS, Dai M, Li L (2018). Circular RNAs mediated by transposons are associated with transcriptomic and phenotypic variation in maize. New Phytol.

[CR7] Cheng EC, Lin H (2013). Repressing the repressor: a lincRNA as a MicroRNA sponge in embryonic stem cell self-renewal. Dev Cell.

[CR8] Dai X, Zhuang Z, Zhao PX (2018). psRNATarget: a plant small RNA target analysis server (2017 release). Nucleic Acids Res.

[CR9] Du H, Yu Y, Ma Y, Gao Q, Cao Y, Chen Z, Ma B, Qi M, Li Y, Zhao X, Wang J, Liu K, Qin P, Yang X, Zhu L, Li S, Liang C (2017). Sequencing and de novo assembly of a near complete indica rice genome. Nat Commun.

[CR10] El-Gebali S, Mistry J, Bateman A, Eddy SR, Luciani A, Potter SC, Qureshi M, Richardson LJ, Salazar GA, Smart A, Sonnhammer ELL, Hirsh L, Paladin L, Piovesan D, Tosatto SCE, Finn RD (2019). The Pfam protein families database in 2019. Nucleic Acids Res.

[CR11] Eren H, Pekmezci MY, Okay S, Turktas M, Inal B, Ilhan E, Atak M, Erayman M, Unver T (2015). Hexaploid wheat (Triticum aestivum) root miRNome analysis in response to salt stress. Ann Appl Biol.

[CR12] Ferreira HJ, Davalos V, de Moura MC, Soler M, Perez-Salvia M, Bueno-Costa A, Setien F, Moran S, Villanueva A, Esteller M (2018). Circular RNA CpG island hypermethylation-associated silencing in human cancer. Oncotarget.

[CR13] Fojtova M, Kovarik A, Matyasek R (2001). Cytosine methylation of plastid genome in higher plants. Fact or artefact?. Plant Sci.

[CR14] Gao Y, Wang J, Zhao F (2015). CIRI: an efficient and unbiased algorithm for de novo circular RNA identification. Genome Biol.

[CR15] Hansen TB, Veno MT, Damgaard CK, Kjems J (2016). Comparison of circular RNA prediction tools. Nucleic Acids Res.

[CR16] Katoh K, Misawa K, Kuma K, Miyata T (2002). MAFFT: a novel method for rapid multiple sequence alignment based on fast Fourier transform. Nucleic Acids Res.

[CR17] Kim D, Landmead B, Salzberg SL (2015). HISAT: a fast spliced aligner with low memory requirements. Nat Methods.

[CR18] Kindgren P, Ard R, Ivanov M, Marquardt S (2018). Transcriptional read-through of the long non-coding RNA SVALKA governs plant cold acclimation. Nat Commun.

[CR19] Law JA, Jacobsen SE (2010). Establishing, maintaining and modifying DNA methylation patterns in plants and animals. Nat Rev Genet.

[CR20] Li L, Stoeckert CJ, Roos DS (2003). OrthoMCL: identification of ortholog groups for eukaryotic genomes. Genome Res.

[CR21] Liu J, Wang H, Chua NH (2015). Long noncoding RNA transcriptome of plants. Plant Biotechnol J.

[CR22] Love MI, Huber W, Anders S (2014). Moderated estimation of fold change and dispersion for RNA-seq data with DESeq2. Genome Biol.

[CR23] Lu T, Cui L, Zhou Y, Zhu C, Fan D, Gong H, Zhao Q, Zhou C, Zhao Y, Lu D, Luo J, Wang Y, Tian Q, Feng Q, Huang T, Han B (2015). Transcriptome-wide investigation of circular RNAs in rice. RNA.

[CR24] Lv W, Jin J, Xu Z, Luo H, Guo Y, Wang X, Wang S, Zhang J, Zuo H, Bai W, Peng Y, Tang J, Zhao S, Zuo B (2020) lncMGPF is a novel positive regulator of muscle growth and regeneration. J Cachexia Sarcopenia Muscle. 10.1002/jcsm.12623 10.1002/jcsm.12623 PMC7749533 32954689

[CR25] Mehrotra S, Goyal V (2014). Repetitive sequences in plant nuclear DNA: types, distribution, evolution and function. Genomics Proteomics Bioinformatics.

[CR26] Memczak S, Jens M, Elefsinioti A, Torti F, Krueger J, Rybak A, Maier L, Mackowiak SD, Gregersen LH, Munschauer M, Loewer A, Ziebold U, Landthaler M, Kocks C, le Noble F, Rajewsky N (2013). Circular RNAs are a large class of animal RNAs with regulatory potency. Nature.

[CR27] Meng X, Zhang P, Chen Q, Wang J, Chen M (2018). Identification and characterization of ncRNA-associated ceRNA networks in Arabidopsis leaf development. BMC Genomics.

[CR28] Miura K, Ikeda M, Matsubara A, Song XJ, Ito M, Asano K, Matsuoka M, Kitano H, Ashikari M (2010). OsSPL14 promotes panicle branching and higher grain productivity in rice. Nat Genet.

[CR29] Morris KV, Mattick JS (2014). The rise of regulatory RNA. Nat Rev Genet.

[CR30] O'Brien J, Hayder H, Zayed Y, Peng C (2018). Overview of MicroRNA biogenesis, mechanisms of actions, and circulation. Front Endocrinol (Lausanne).

[CR31] Shin SY, Jeong JS, Lim JY, Kim T, Park JH, Kim JK, Shin C (2018) Transcriptomic analyses of rice (Oryza sativa) genes and non-coding RNAs under nitrogen starvation using multiple omics technologies. BMC Genomics 19. 10.1186/s12864-018-4897-1 10.1186/s12864-018-4897-1 PMC6043990 30005603

[CR32] Singh U, Khemka N, Rajkumar MS, Garg R, Jain M (2017) PLncPRO for prediction of long non-coding RNAs (lncRNAs) in plants and its application for discovery of abiotic stress-responsive lncRNAs in rice and chickpea. Nucleic Acids Res 45(22). 10.1093/nar/gkx866 10.1093/nar/gkx866 PMC5727461 29036354

[CR33] Song JM, Lei Y, Shu CC, Ding Y, Xing F, Liu H, Wang J, Xie W, Zhang J, Chen LL (2018). Rice information GateWay: a comprehensive bioinformatics platform for Indica Rice genomes. Mol Plant.

[CR34] Stamatakis A (2014). RAxML version 8: a tool for phylogenetic analysis and post-analysis of large phylogenies. Bioinformatics.

[CR35] Sun XY, Wang L, Ding JC, Wang YR, Wang JS, Zhang XY, Che YL, Liu ZW, Zhang XR, Ye JZ, Wang J, Sablok G, Deng ZP, Zhao HW (2016). Integrative analysis of Arabidopsis thaliana transcriptomics reveals intuitive splicing mechanism for circular RNA. FEBS Lett.

[CR36] Trapnell C, Roberts A, Goff L, Pertea G, Kim D, Kelley DR, Pimentel H, Salzberg SL, Rinn JL, Pachter L (2012). Differential gene and transcript expression analysis of RNA-seq experiments with TopHat and cufflinks. Nat Protoc.

[CR37] Wang H, Chung PJ, Liu J, Jang IC, Kean MJ, Xu J, Chua NH (2014). Genome-wide identification of long noncoding natural antisense transcripts and their responses to light in Arabidopsis. Genome Res.

[CR38] Wang H, Jiao X, Kong X, Hamera S, Wu Y, Chen X, Fang R, Yan Y (2016) A Signaling Cascade from miR444 to RDR1 in Rice Antiviral RNA Silencing Pathway. Plant Physiol 170(4):2365-77. . 10.1104/pp.15.01283 PMC4825140 26858364

[CR39] Wang HLV, Chekanova JA (2017). Long noncoding RNAs in plants. Adv Exp Med Biol.

[CR40] Wang J, Ye Y, Xu M, Feng L, Xu L (2019). Roles of the SPL gene family and miR156 in the salt stress responses of tamarisk (Tamarix chinensis). BMC Plant Biology.

[CR41] Wang Y, Luo X, Sun F, Hu J, Zha X, Su W, Yang J (2018). Overexpressing lncRNA LAIR increases grain yield and regulates neighbouring gene cluster expression in rice. Nat Commun.

[CR42] Wang Y, Xu Z, Jiang J, Xu C, Kang J, Xiao L, Wu M, Xiong J, Guo X, Liu H (2013). Endogenous miRNA sponge lincRNA-RoR regulates Oct4, Nanog, and Sox2 in human embryonic stem cell self-renewal. Dev Cell.

[CR43] Wei LY, Gu LF, Song XW, Cui XK, Lu ZK, Zhou M, Wang LL, Hu FY, Zhai JX, Meyers BC, Cao XF (2014). Dicer-like 3 produces transposable element-associated 24-nt siRNAs that control agricultural traits in rice. P Natl Acad Sci USA.

[CR44] Whalen S, Truty RM, Pollard KS (2016). Enhancer-promoter interactions are encoded by complex genomic signatures on looping chromatin. Nat Genet.

[CR45] Wucher V, Legeai F, Hedan B, Rizk G, Lagoutte L, Leeb T, Jagannathan V, Cadieu E, David A, Lohi H, Cirera S, Fredholm M, Botherel N, Leegwater PAJ, Le Beguec C, Fieten H, Johnson J, Alfoldi J, Andre C, Lindblad-Toh K, Hitte C, Derrien T (2017) FEELnc: a tool for long non-coding RNA annotation and its application to the dog transcriptome. Nucleic Acids Res 45(8). 10.1093/nar/gkw1306 10.1093/nar/gkw1306 PMC5416892 28053114

[CR46] Xu W, Yang TQ, Wang B, Han B, Zhou HK, Wang Y, Li DZ, Liu AZ (2018). Differential expression networks and inheritance patterns of long non-coding RNAs in castor bean seeds. Plant J.

[CR47] Yan Y, Wang H, Hamera S et al (2014) Mir444a has multiple functions in the rice nitrate-signaling pathway. Plant J. 10.1111/tpj.12446 10.1111/tpj.12446 24460537

[CR48] Zhang JW, Chen LL, Xing F, Kudrna DA, Yao W, Copetti D, Mu T, Li WM, Song JM, Xie WB, Lee S, Talag J, Shao L, An Y, Zhang CL, Ouyang YD, Sun S, Jiao WB, Lv F, Du BG, Luo MZ, Maldonado CE, Goicoechea JL, Xiong LZ, Wu CY, Xing YZ, Zhou DX, Yu SB, Zhao Y, Wang GW, Yu YS, Luo YJ, Zhou ZW, Hurtado BEP, Danowitz A, Wing RA, Zhang QF (2016). Extensive sequence divergence between the reference genomes of two elite indica rice varieties Zhenshan 97 and Minghui 63. P Natl Acad Sci USA.

[CR49] Zhang XO, Dong R, Zhang Y, Zhang JL, Luo Z, Zhang J, Chen LL, Yang L (2016). Diverse alternative back-splicing and alternative splicing landscape of circular RNAs. Genome Res.

[CR50] Zhang ZH, Yu JY, Li DF, Zhang ZY, Liu FX, Zhou X, Wang T, Ling Y, Su Z (2010). PMRD: plant microRNA database. Nucleic Acids Res.

[CR51] Zhao L, Wang SQ, Cao ZL, Ouyang WZ, Zhang Q, Xie L, Zheng RQ, Guo MR, Ma M, Hu Z, Sung WK, Zhang QF, Li GL, Li XW (2019). Chromatin loops associated with active genes and heterochromatin shape rice genome architecture for transcriptional regulation Nature Communications.

[CR52] Zhao L, Xie L, Zhang Q, Ouyang W, Deng L, Guan P, Ma M, Li Y, Zhang Y, Xiao Q, Zhang J, Li H, Wang S, Man J, Cao Z, Zhang Q, Zhang Q, Li G, Li X (2020). Integrative analysis of reference epigenomes in 20 rice varieties. Nat Commun.

[CR53] Zhao T, Tao X, Feng S, Wang L, Hong H, Ma W, Shang G, Guo S, He Y, Zhou B, Guan X (2018). LncRNAs in polyploid cotton interspecific hybrids are derived from transposon neofunctionalization. Genome Biol.

[CR54] Zhong S, Fei Z, Chen YR, Zheng Y, Huang M, Vrebalov J, McQuinn R, Gapper N, Liu B, Xiang J, Shao Y, Giovannoni JJ (2013). Single-base resolution methylomes of tomato fruit development reveal epigenome modifications associated with ripening. Nat Biotechnol.

