# Supplementary material for: Analysis of Rice Transcriptome Reveals the LncRNA/CircRNA Regulation in Tissue Development
Source: Rice (N Y). 2021 Jan 28;14:14. doi: 10.1186/s12284-021-00455-2 (PMC7843763; doi:10.1186/s12284-021-00455-2)
Supplement: Supplementary file 2 — Additional file 2: Figure S1. Workflow and characteristics of lncRNAs and circRNAs in rice. Figure S2. qPCR of 27 circRNAs from young leaf of MH63. Figure S3. DNA methylation of P-circRNAs and lncRNA loci in ZS97. Figure S4. Comprehensive epigenome map of lncRNA and P-circRNA in different varieties. Figure S5. Tissue specificity of lncRNAs and circRNAs in MH63. Figure S6. Gene ontology analysis of the target genes in MH63. Figure S7. Luciferase reporter assays of lncRNAs in 297 T cells. Figure S8. CeRNA network of DECs and DELs of panicle in MH63. Figure S9. CeRNA network of DECs and DELs of seedling in MH63. Figure S10. CeRNA network of DECs and DELs of root in MH63. [file 12284_2021_455_MOESM2_ESM.pdf]

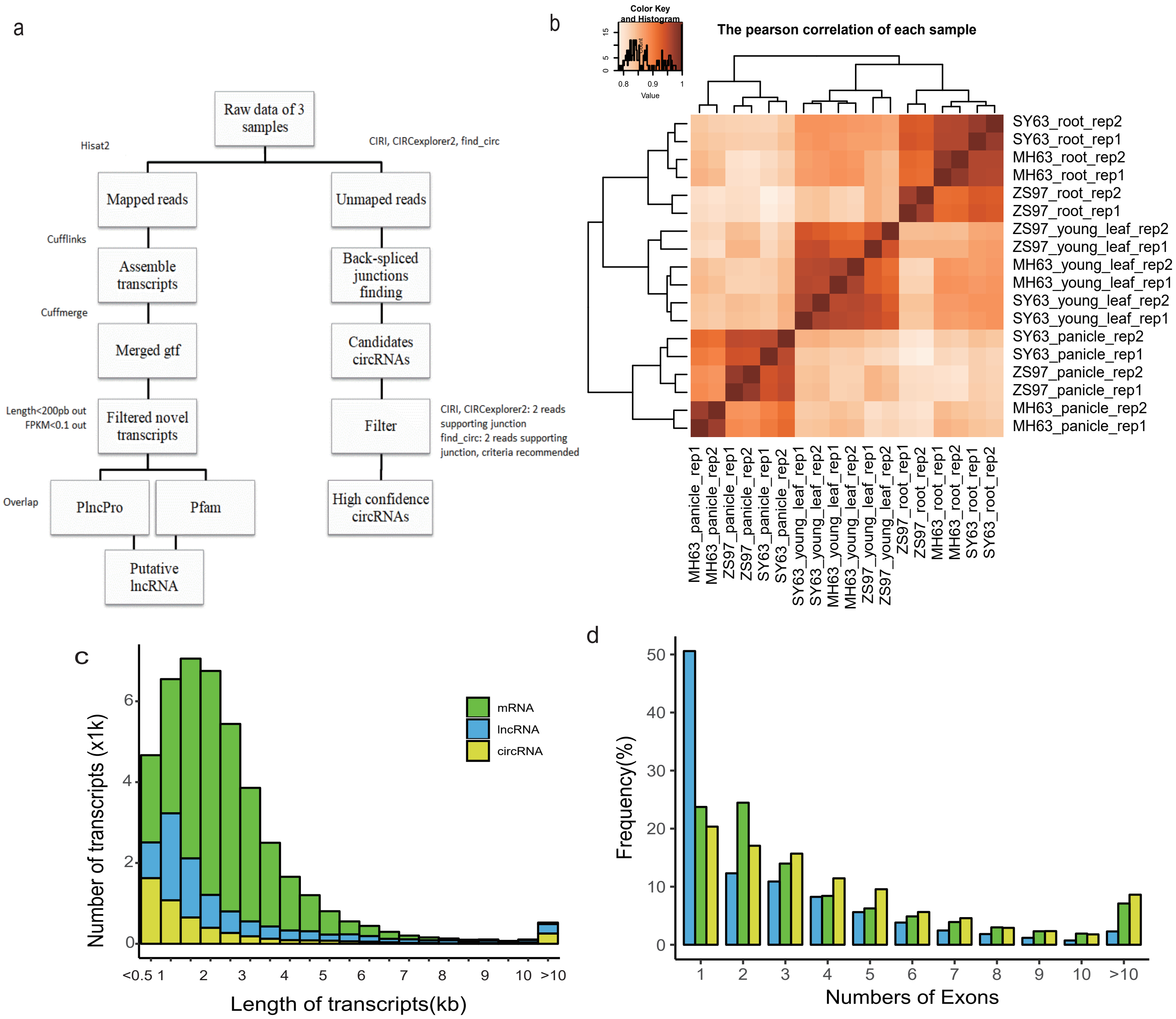

Figure S1. Workflow and characteristics of lncRNAs and circRNAs in rice. (a) Computational identification of lncRNAs and circRNAs. (b)The Pearson's correlation of different samples. The distribution of exon number for circRNAs, lncRNAs and mRNAs in rice. (c,d) Length and number of exons of lncRNAs, PC genes and circRNAs in MH63.

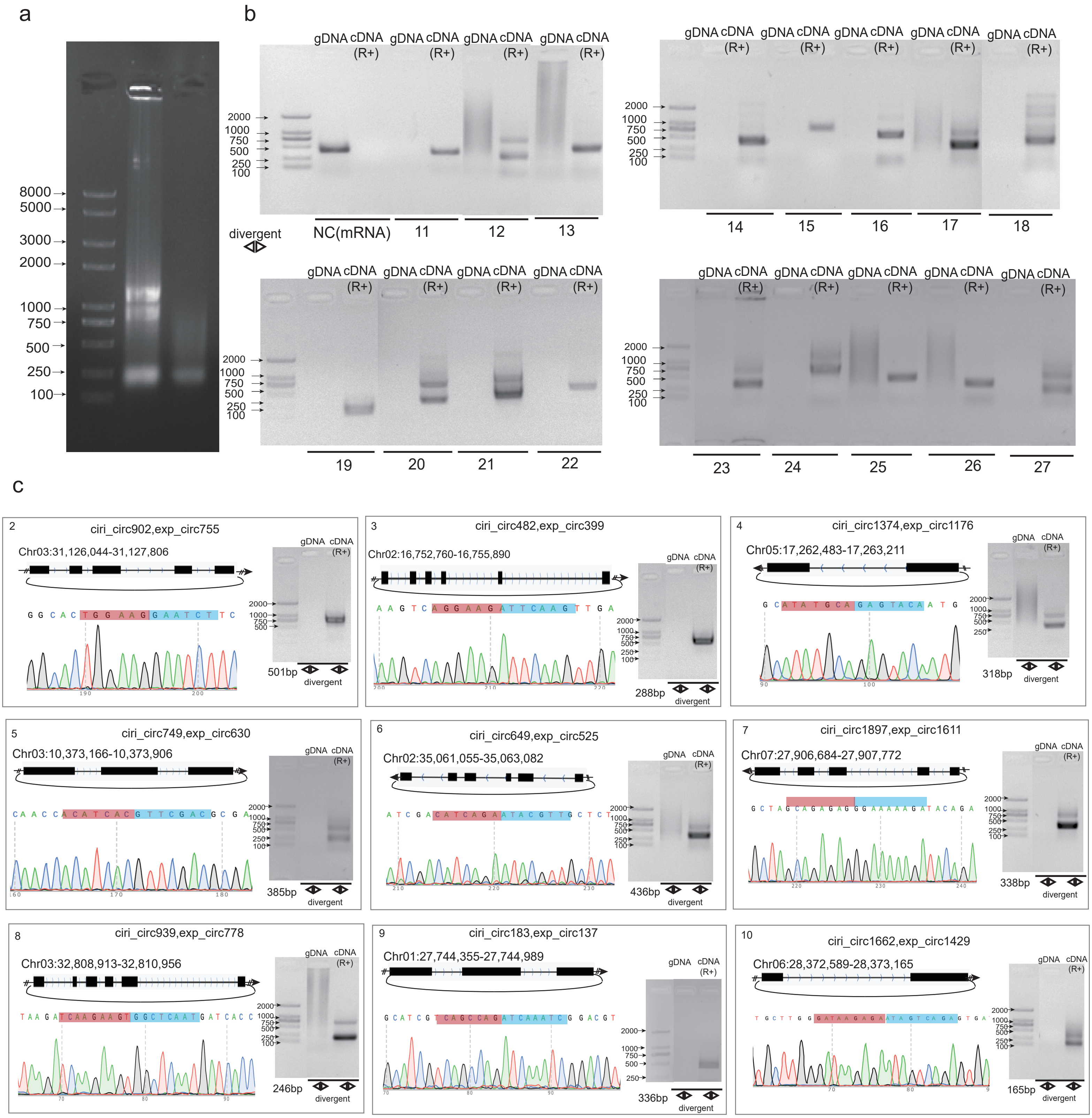

Figure S2. qPCR of 27 circRNAs from young leaf of MH63. (a) Electrophoresis of total RNA and RNA treated with RNaseR (b) qPCR of No. 11~27 circRNAs that were successfully amplified. (c) The No. 2~10 of CircRNAs that were successfully amplified and sequenced for the validation. R+, represent samples with RNase R treatment. The black dotted line represents the splice site.

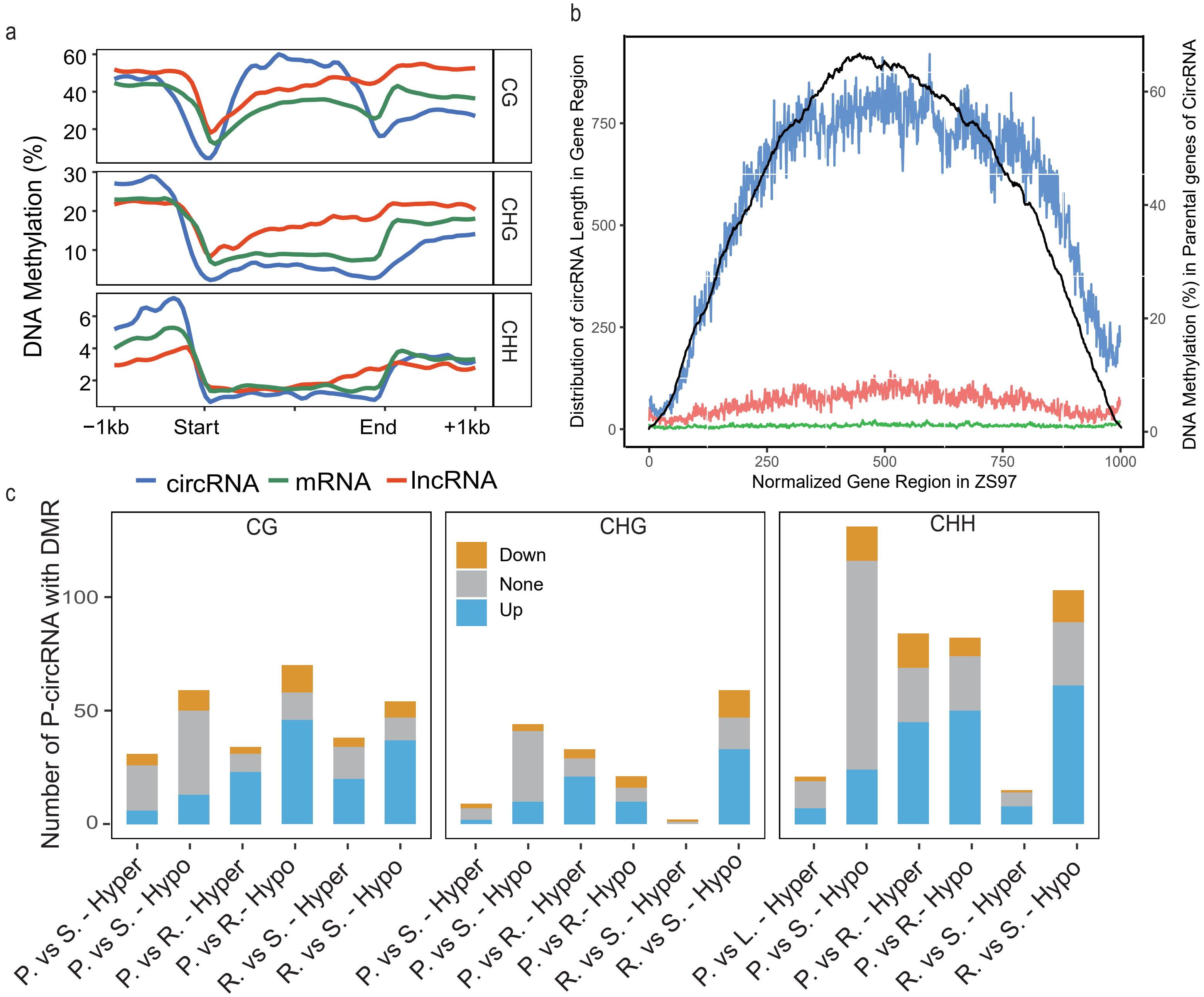

Figure S3. DNA methylation of P-circRNAs and lncRNA loci in ZS97. (a) CG, CHG and CHH DNA methylation densities in the parental genes of circRNAs (blue), lncRNAs locus (red) and PC genes (green) and their 1 kb up/down flanking regions in seedling of ZS97. (b) Distribution of the positions of circRNAs (black) and CG DNA methylation (red) in the parental genes normalized to 1 kb length in ZS97. (c) Histogram of P-circRNA containing hyper- and hypo-methylation on DMRs in body. P-circRNAs were classified into the same groups as in (a). DNA methylation (CG, CHG and CHH) profile of non-differently expressed (None, grey), upregulated (Up, orange), and downregulated (Down, blue) P-circRNAs in MH63. P. vs S. referred to the comparison between panicle and seedling. P. vs R. referred to the comparison between panicle and root. R. vs. S. referred to the comparison between root and seedling.

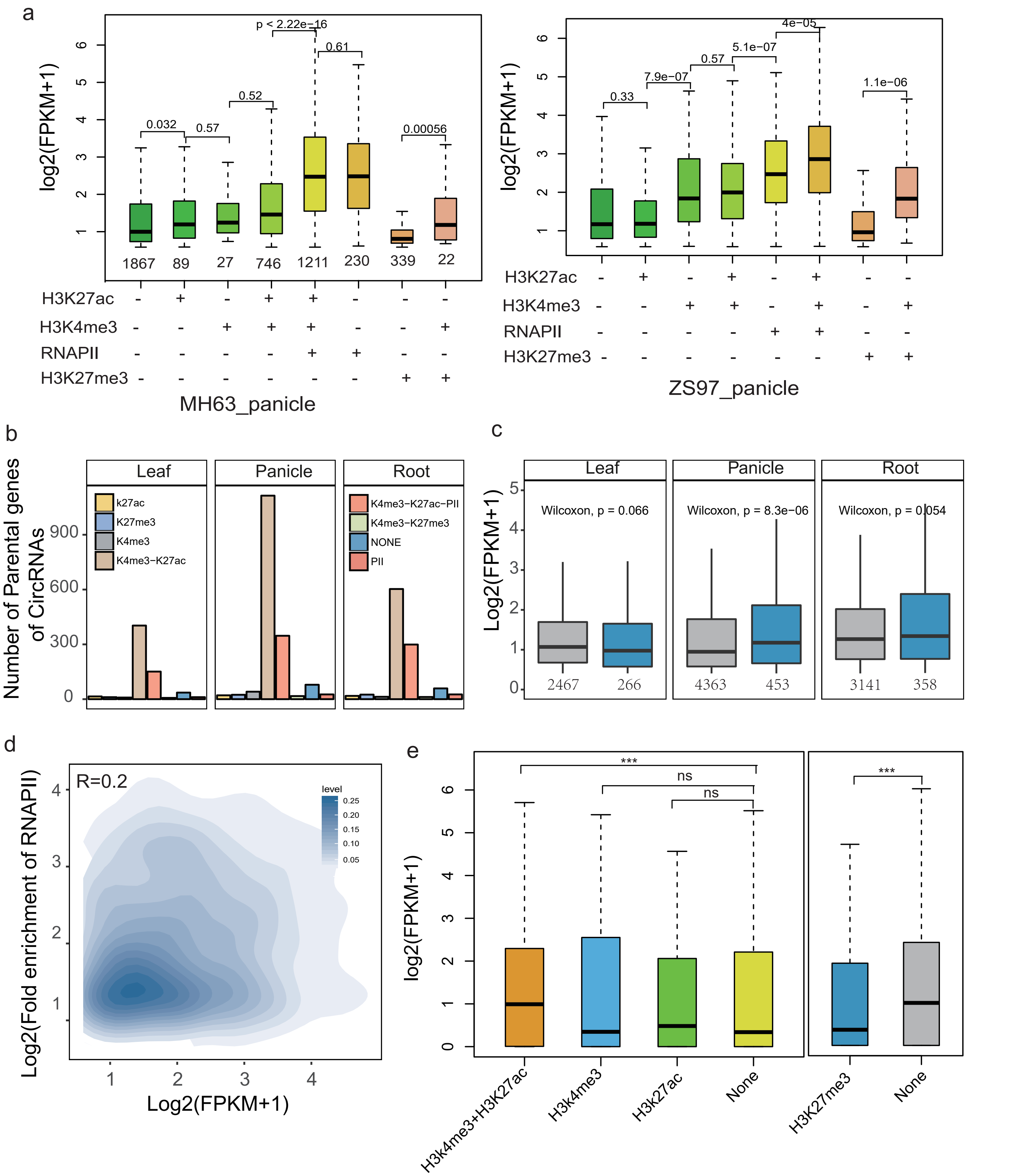

Figure S4. Comprehensive epigenome map of lncRNA and P-circRNA in different varieties. (a) Expression levels of lncRNA loci marked by different histone modifications and RNAPII in panicle of MH63 (left) and ZS97 (right). Numbers of locus with histones categories were shown. (b) Numbers of P-circRNA marked by different histone modifications and RNAPII of leaf (left), panicle (middle) and root (right) in MH63. (c) Comparison of the number and expression (FPKM) of lncRNAs marked or not-marked by H3K9me2. (d) The relationship between intensity of RNAPII and expression of lncRNA loci in seedling of MH63. (d) Expression levels of genes nearest lncRNA loci marked by RNAPII and histone modification. Wilcoxon's test was used for all the tests in the figures, *** means P <0.001, ns means no significance.

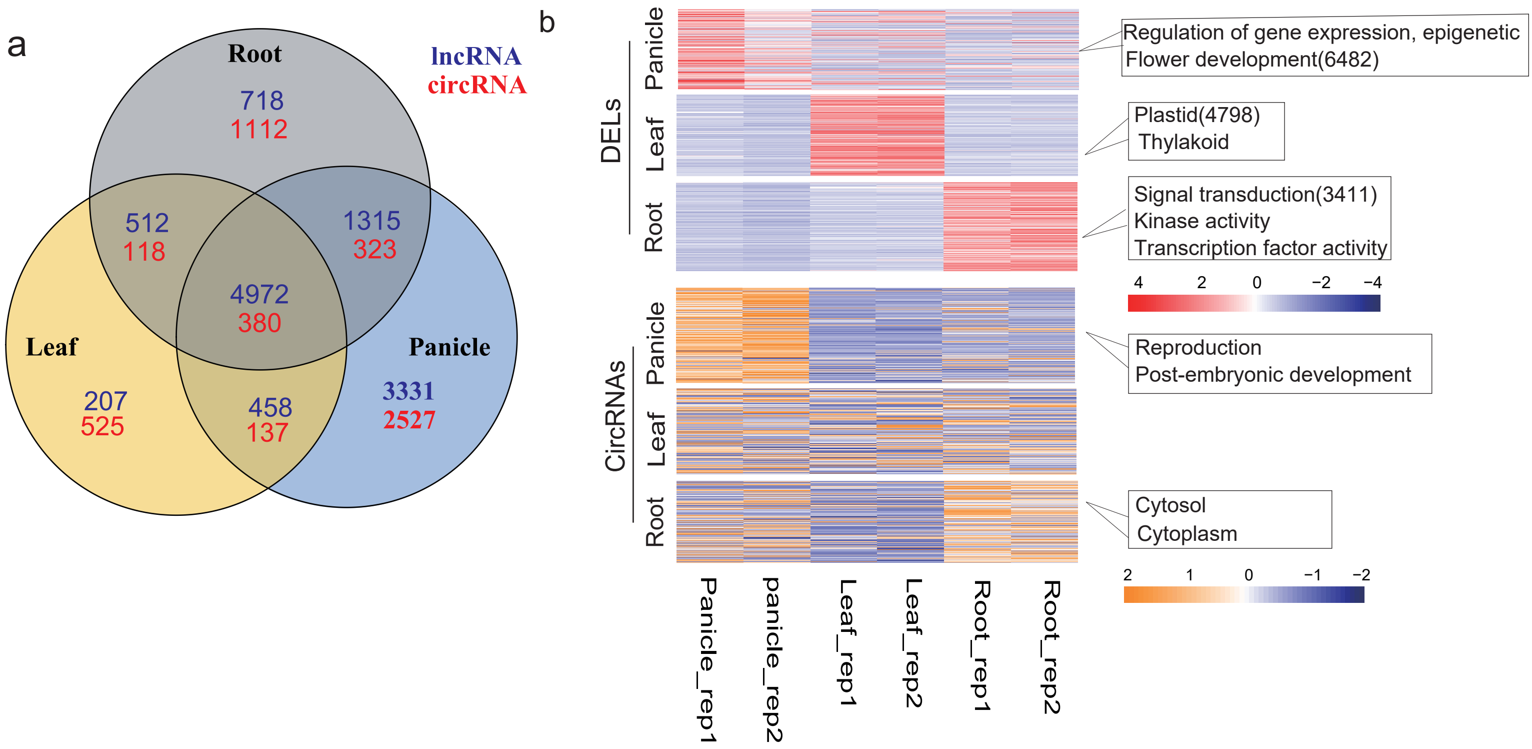

Figure S5. Tissue specificity of lncRNAs and circRNAs in MH63. (a) Number of lncRNAs and circRNAs in different tissues. (b) Differential expression analysis of lncRNAs and circRNAs in different tissues and replicates of MH63, and the results of GO analysis in each tissue.

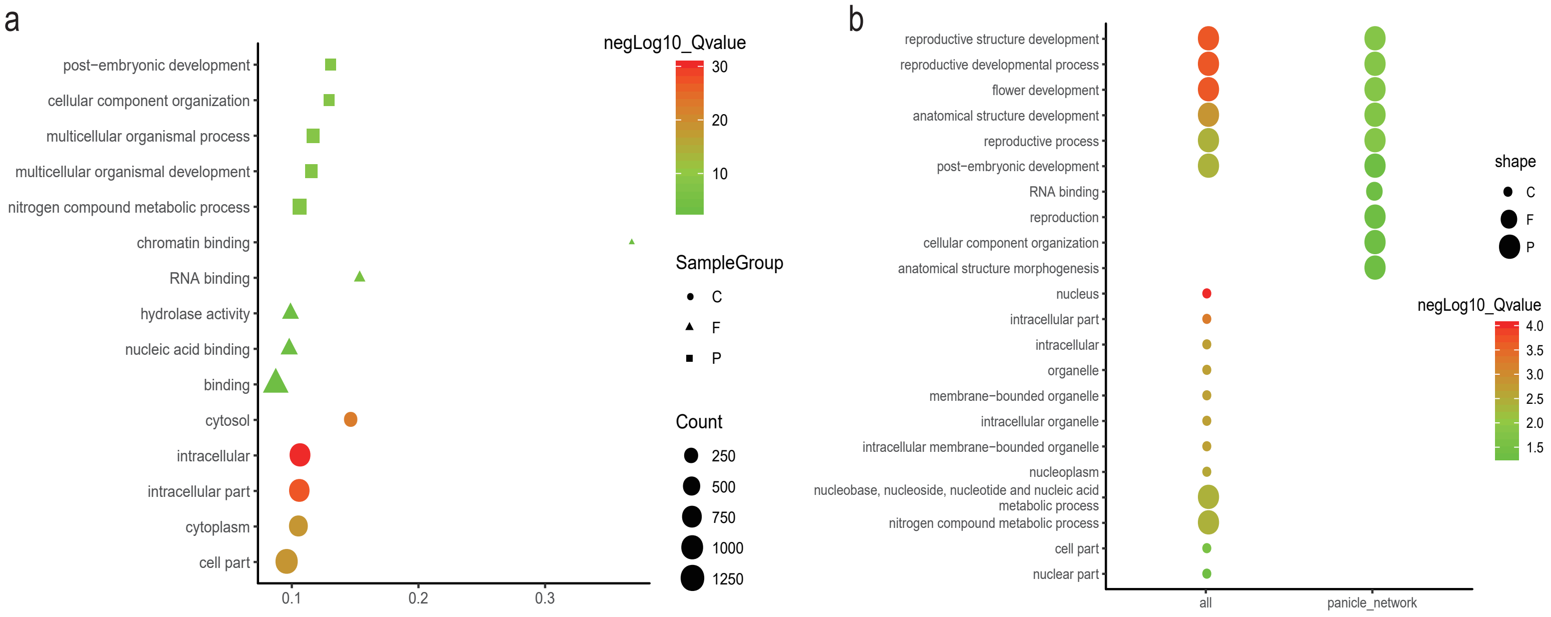

Figure S6. Gene ontology analysis of the target genes in MH63. (a) GO of parental genes of circRNAs. GO terms are summarized in three categories: cellular component (C), molecular function (F) and biological process (P). (b) Gene ontology analysis of with the target genes of miRNAs in the ceRNA networks in MH63. GO terms are summarized in three categories: cellular component (C), molecular function (F) and biological process (P).

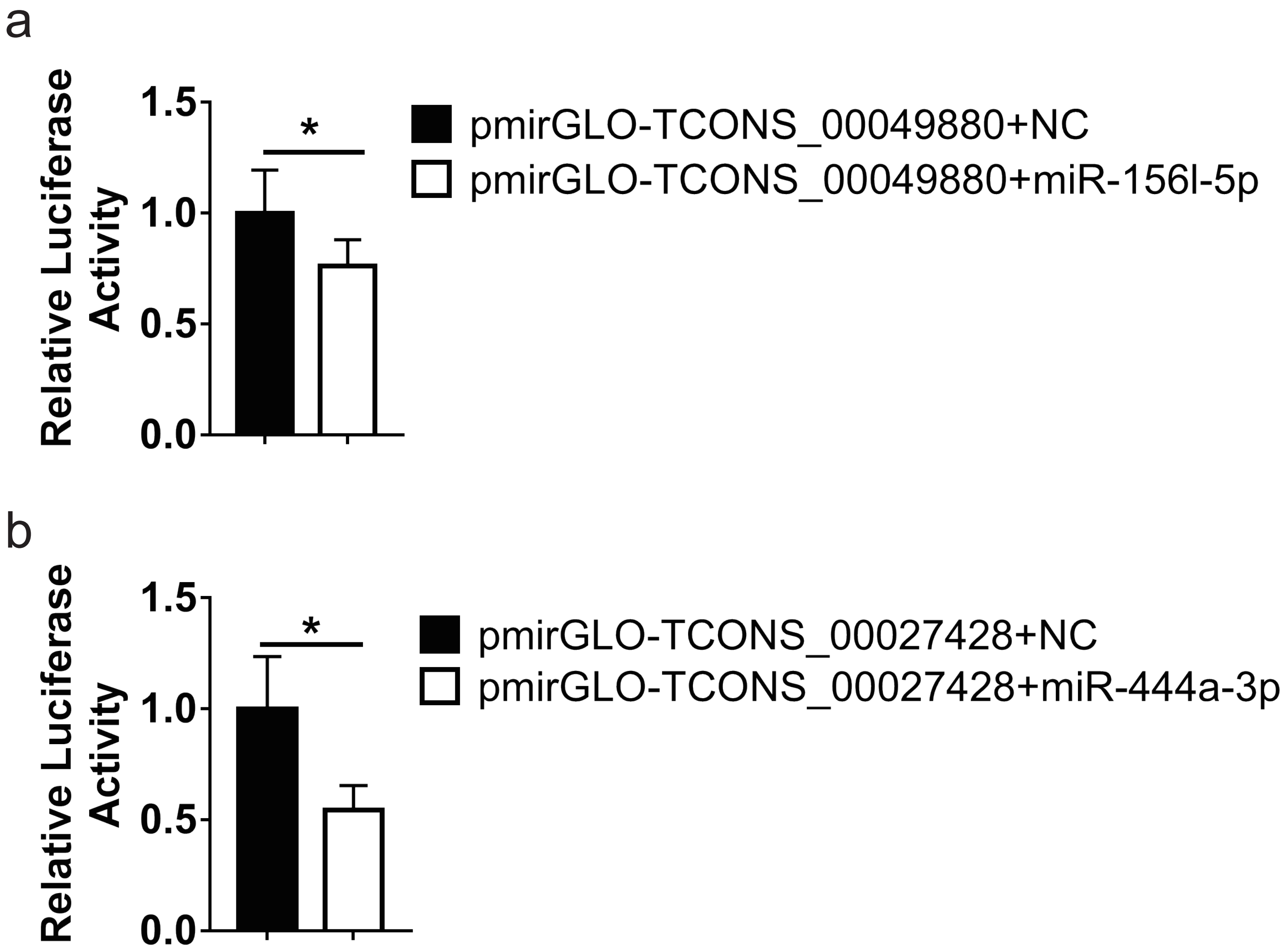

Figure S7. Luciferase reporter assays of lncRNAs in 293T cells. Luciferase reporter assays of lncRNA TCONS_00049880 and lncRNA TCONS_00027428 in luciferase construct in 293T cells showed that osa-miR156l-5p specifically targets TCONS_000049880 and osa-miR444a-3p specifically targets TCONS_00027428.

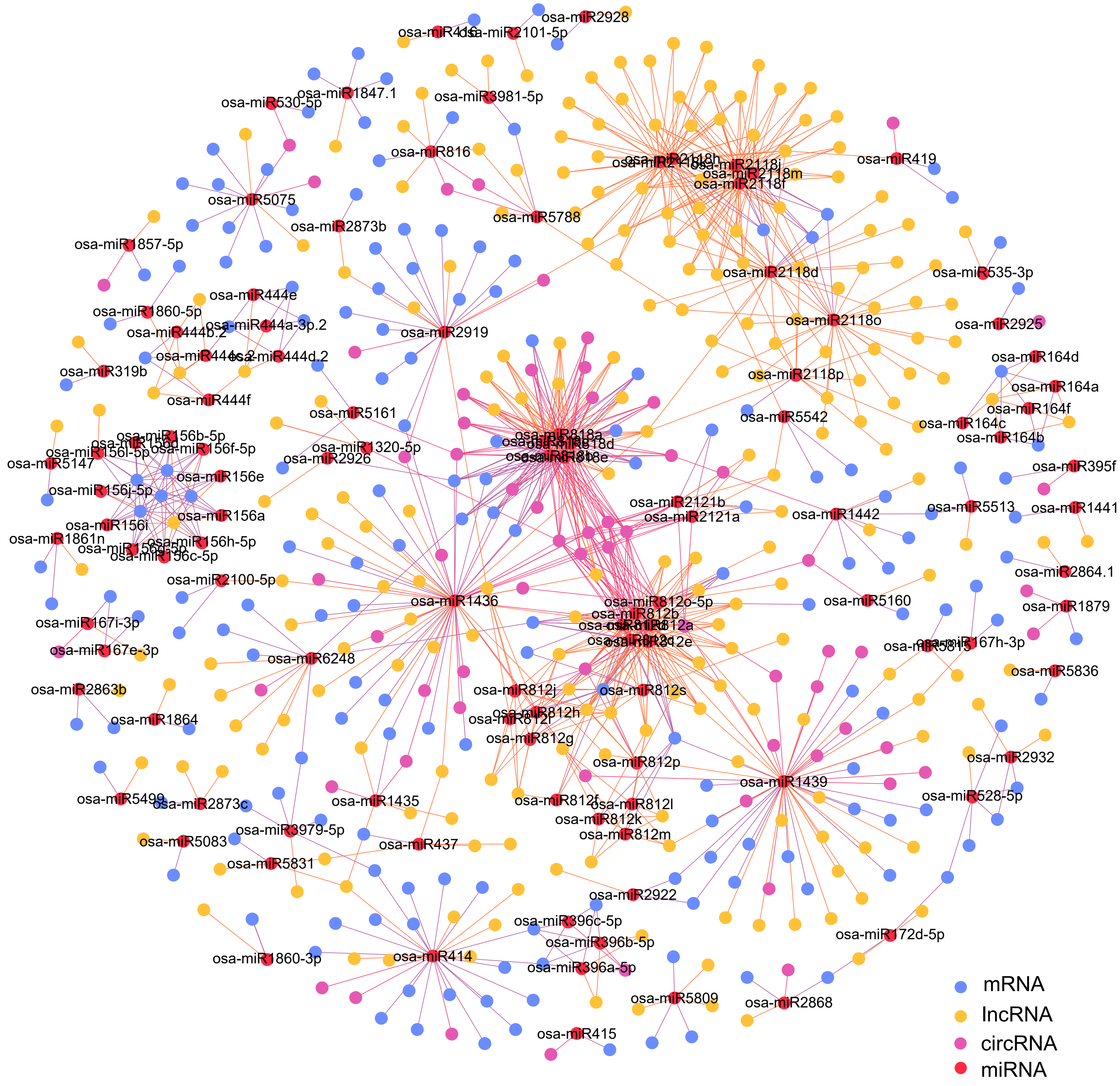

Figure S8. CeRNA network of DECs and DELs of panicle in MH63. Each color represents different interactions of a miRNA in the ceRNA network.

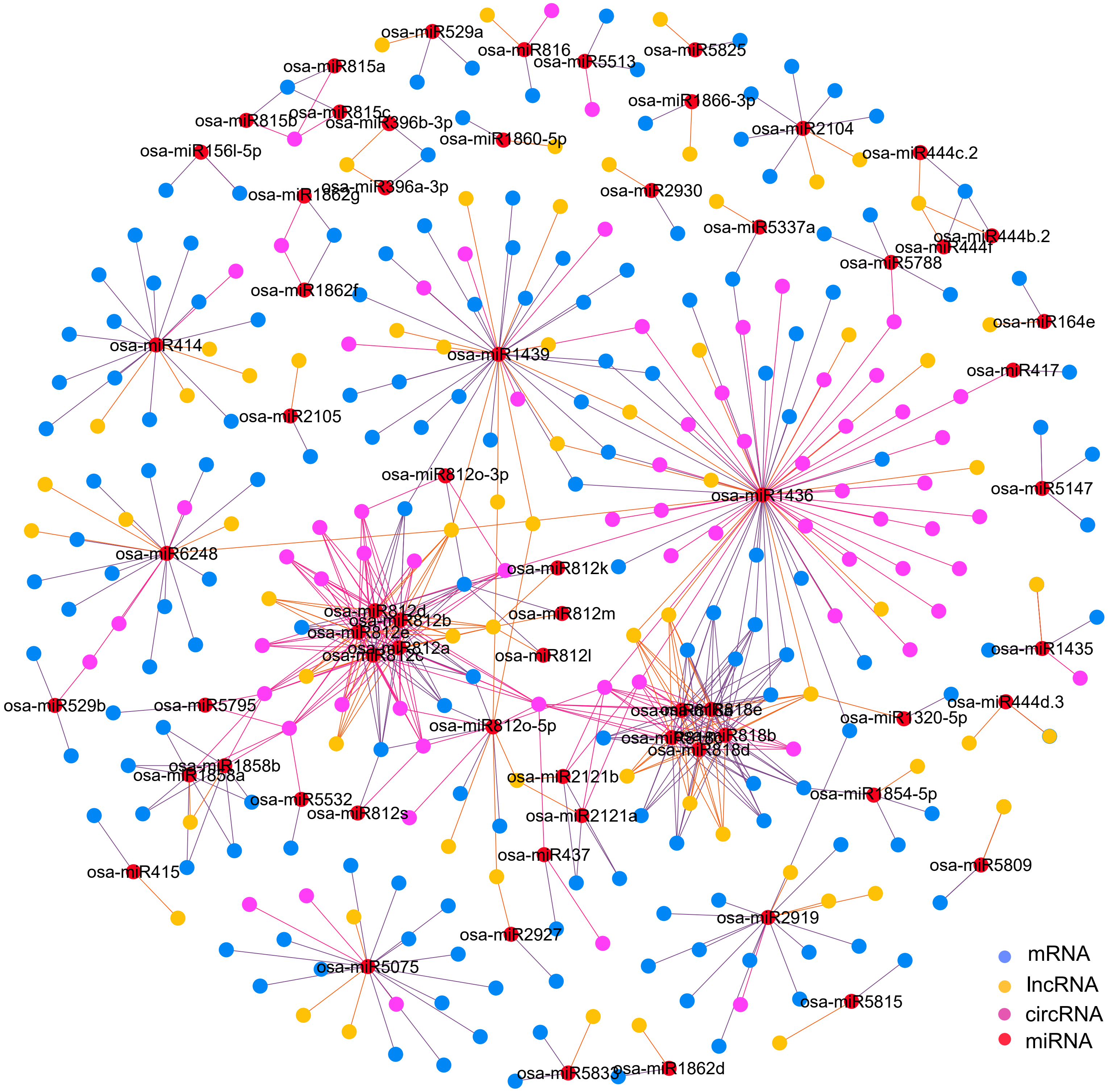

Figure S9. CeRNA network of DECs and DELs of seedling in MH63. Each color represents different interactions of a miRNA in the ceRNA network.

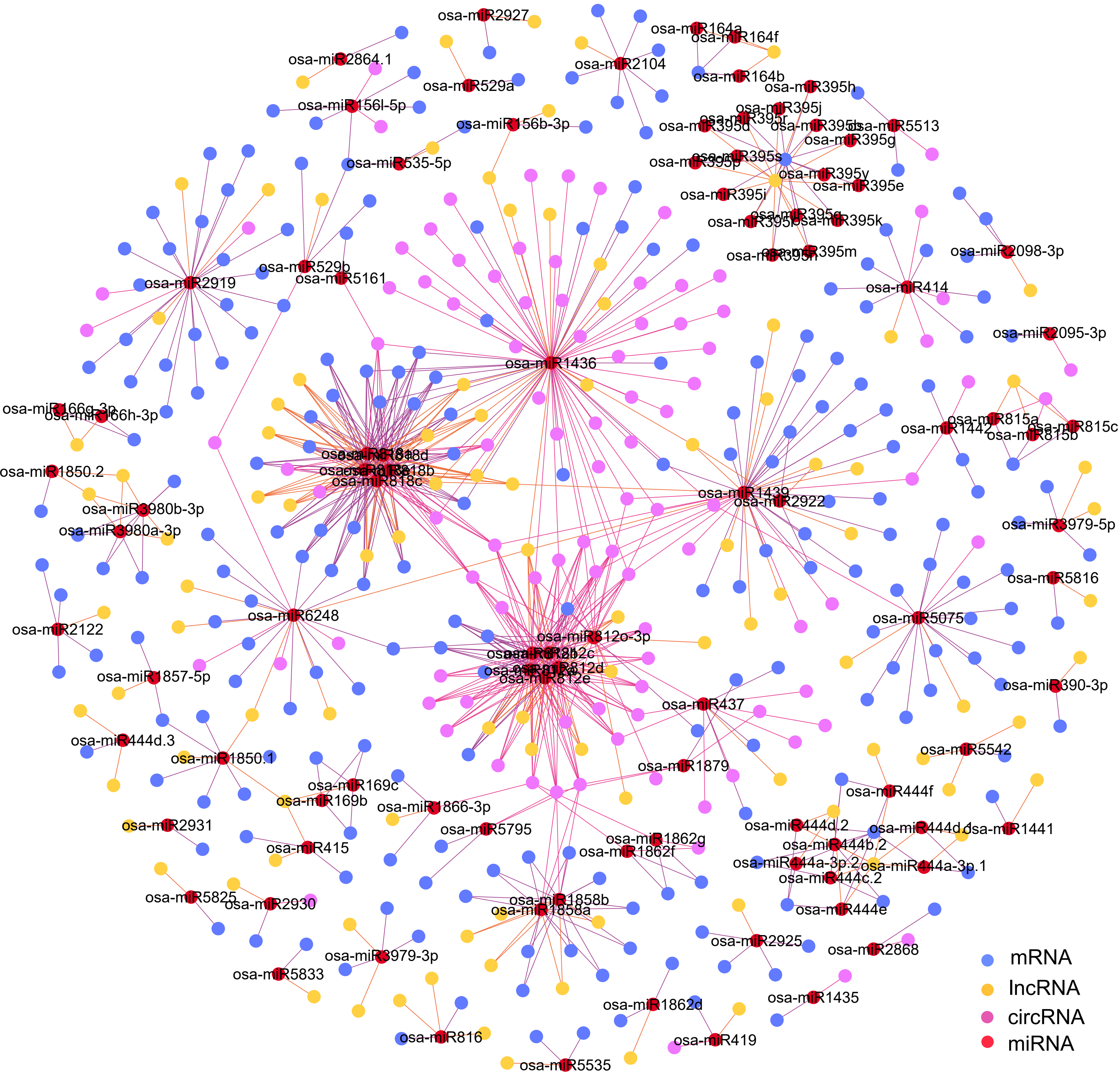

Figure S10. CeRA network of DECs and DELs of root in MH63. Each color represents the different interactions of a miRNA in the ceRNA network.
