# Supplementary material for: Analysis of Rice Transcriptome Reveals the LncRNA/CircRNA Regulation in Tissue Development
Source: Rice (N Y). 2021 Jan 28;14:14. doi: 10.1186/s12284-021-00455-2 (PMC7843763; doi:10.1186/s12284-021-00455-2)
Supplement: Supplementary file 3 — Additional file 3: Table S1. Data of RNA-seq in this study. Table S2. Tissue distribution of ncRNAs. Table S3. Replicate distribution of circRNAs. Table S4. Software distribution of high-confidence circRNAs. Table S5. Primers list of 27 validated circRNAs. Table S6. Alternative splicing of circRNAs. Table S7. Genomic distribution of circRNAs. Table S8. Genomic distribution of lncRNAs. Table S9. Conservation of lncRNAs and circRNAs in MH63 with other species. Table S10. The proportion of number/length attributable to different TE superfamilies of TE-related lncRNAs/circRNAs. [file 12284_2021_455_MOESM3_ESM.doc]

# Supplementary Tables and Figures

**Table S1: Data of RNA-seq in this study**

| **Sample** | **Raw**  **Reads** | **Clean Reads** | **Error Rate (%)** | **Q20(%)** | **Q30(%)** | **GC content (%)** | **Coverage** |
| --- | --- | --- | --- | --- | --- | --- | --- |
| MH-panicle-rep1 | 69,750,648 | 69,028,601 | 0.01 | 96.68 | 91.81 | 49.02 | 53x |
| MH-panicle-rep2 | 66,898,732 | 66,244,106 | 0.01 | 96.75 | 91.94 | 49.59 | 52x |
| MH-young_leaf-rep1 | 69,972,520 | 69,328,773 | 0.03 | 97.49 | 93.48 | 45.78 | 53x |
| MH-young_leaf-rep2 | 68,017,224 | 67,357,457 | 0.03 | 97.24 | 92.94 | 45.91 | 52x |
| MH-root-rep1 | 71,355,101 | 70,353,068 | 0.01 | 97.00 | 92.50 | 49.60 | 54x |
| MH-root-rep2 | 81,017,424 | 79,729,247 | 0.02 | 94.78 | 88.39 | 49.48 | 61x |
| ZS-panicle-rep1 | 68,494,988 | 68,028,011 | 0.01 | 96.59 | 91.63 | 48.59 | 52x |
| ZS-panicle-rep2 | 75,335,387 | 74,559,808 | 0.01 | 96.89 | 92.29 | 50.31 | 57x |
| ZS-young_leaf-rep1 | 74,849,106 | 74,305,617 | 0.03 | 97.34 | 93.19 | 47.22 | 57x |
| ZS-young_leaf-rep2 | 72,444,163 | 71,844,388 | 0.03 | 97.38 | 93.26 | 47.45 | 55x |
| ZS-root-rep1 | 72,320,671 | 71,174,967 | 0.01 | 97.59 | 93.70 | 50.72 | 54x |
| ZS-root-rep2 | 76,087,567 | 75,077,463 | 0.01 | 97.45 | 93.47 | 50.36 | 57x |
| SY-panicle-rep1 | 76,735,606 | 76,101,702 | 0.01 | 96.90 | 92.37 | 48.61 | 58x |
| SY-panicle-rep2 | 70,934,603 | 70,310,723 | 0.01 | 96.71 | 91.94 | 49.47 | 54x |
| SY-young_leaf-rep1 | 71,070,379 | 70,423,639 | 0.03 | 97.39 | 93.30 | 45.78 | 54x |
| SY-young_leaf-rep2 | 68,095,109 | 67,775,062 | 0.03 | 97.09 | 92.69 | 45.48 | 52x |
| SY-root-rep1 | 71,007,755 | 69,860,766 | 0.01 | 97.50 | 93.62 | 48.95 | 53x |
| SY-root-rep2 | 82,370,593 | 81,022,123 | 0.01 | 97.63 | 93.87 | 49.48 | 62x |

**Table S2: Tissue distribution of ncRNAs**

| Tissue | circRNAs | | | lncRNAs | | |
| --- | --- | --- | --- | --- | --- | --- |
|  | *MH63* | *ZS97* | *SY63* | *MH63* | *ZS97* | *SY63* |
| Panicle | 3367 | 2999 | 2898 | 10076 | 11652 | 12028 |
| Leaf | 1160 | 977 | 1173 | 6149 | 6856 | 7185 |
| Root | 1933 | 1245 | 1918 | 7517 | 8720 | 8870 |
| Total | 5122 | 4273 | 4707 | 11,513 | 13,153 | 13,549 |

**Table S3: Replicate distribution of circRNAs**

| Replicates | circRNAs | | |
| --- | --- | --- | --- |
|  | *MH63* | *ZS97* | *SY63* |
| Panicle-rep1 | 1850 | 1407 | 1728 |
| Panicle-rep2 | 1976 | 1886 | 1447 |
| Young_leaf-rep1 | 793 | 638 | 727 |
| Young_leaf-rep2 | 741 | 632 | 756 |
| Root-rep1 | 1169 | 815 | 1024 |
| Root-rep2 | 1143 | 733 | 1181 |

**Table S4: Software distribution of high confident circRNAs**

| Software | CIRI2 | CIRCexplorer2 | find_circ | CIRI2 & CIRCexplorer2 | CIRI2 & find_circ | CIRCexplorer2 & find_circ | CIRI2, CIRCexplorer2 & find_circ |
| --- | --- | --- | --- | --- | --- | --- | --- |
| MH63 | 1383 | 1296 | 1150 | 971 | 296 | 17 | 9 |
| ZS97 | 1044 | 1187 | 1152 | 701 | 164 | 19 | 6 |
| SY63 | 1234 | 1247 | 1203 | 760 | 237 | 17 | 9 |

**Table S5: Primers list of 27 validated circRNAs**

| **No.** | **Name** | **Primer** | **Sequence** | **Size** |
| --- | --- | --- | --- | --- |
| NC | mRNA | Forward | 5‘- CCTCCTGAACACGAGGATCACC-3’ | 461 bp |
|  |  | Reverse | 5‘- CGTTGAACCTGTAGTCGTCGTG-3’ |  |
| 1 | ciri_circ161, exp_circ133 | Primer1 | 5‘- GCTATAGCAGTCAGCATCTCAAG -3’ | 413 bp |
|  |  | Primer2 | 5‘- GGTCTTGGTGAAAGATCTTTTGAGG-3’ |  |
| **2** | ciri_circ902, exp_circ755 | Primer1 | 5‘- CTTCCAGGTTTCAGAGTAATCCTG -3’ | 501 bp |
|  |  | Primer2 | 5‘- GAGTATCACATCATGTCAGAACGC -3’ |  |
| 3 | ciri_circ482, exp_circ399 | Primer1 | 5‘- GTTACTGGAAGGACTTGCTTCCG -3’ | 288 bp |
|  |  | Primer2 | 5‘- GATCGTAACCATGTGACAGTAAAAGGTC -3’ |  |
| 4 | ciri_circ1374, exp_circ1176 | Primer1 | 5‘- CATCTCTGGTGTATTCATCCACC -3’ | 318 bp |
|  |  | Primer2 | 5‘- CGATGAAATGGAACGTGTAGC-3’ |  |
| 5 | ciri_circ749, exp_circ630 | Primer1 | 5‘- GTGTGCGTAGAAGATGTTGCTG -3’ | 385 bp |
|  |  | Primer2 | 5‘- CAACACCTACATCTGGTGGCTC-3’ |  |
| 6 | ciri_circ649, exp_circ525 | Primer1 | 5‘- GTCGACAAGCTCTTTAGTCAAAGG -3’ | 436 bp |
|  |  | Primer2 | 5‘- GGCTCTACAAGCATTTGTCGATC-3’ |  |
| 7 | ciri_circ1897, exp_circ1611 | Primer1 | 5‘- CCCGAGTATAGATGTTGCAGC -3’ | 338 bp |
|  |  | Primer2 | 5‘- CCAGCTTGAAGGCCTAAACTTCC-3’ |  |
| 8 | ciri_circ939, exp_circ778 | Primer1 | 5‘- GATCAATCAATGGTCGTTGCC -3’ | 246 bp |
|  |  | Primer2 | 5‘-GCAAGCCACCTATTCGACCTTC-3’ |  |
| 9 | ciri_circ183, exp_circ137 | Primer1 | 5‘- CTTGAGGTCGCAGTAGCCGAAC-3’ | 336 bp |
|  |  | Primer2 | 5‘- GAGCACGGCGTCTACAACC-3’ |  |
| 10 | ciri_circ1662, exp_circ1429 | Primer1 | 5‘- CTGATGGCGTGCAGCAGTAAC -3’ | 165 bp |
|  |  | Primer2 | 5‘- GGGCAACAGCAGCTCTGAAAC-3’ |  |
| 11 | ciri_circ941, exp_circ783 | Primer1 | 5‘- GAAGTGCCTCGACATGGCCTTC -3’ | 270 bp |
|  |  | Primer2 | 5‘- CATCGACCAGAGCCTCCGG-3’ |  |
| 12 | ciri_circ2121, exp_circ1836 | Primer1 | 5‘- GAGCTTGTCGATCTTGTCGAACAGC -3’ | 290 bp |
|  |  | Primer2 | 5‘- GCAACCAAGTTGTCGGTTCCAG-3’ |  |
| 13 | ciri_circ1164, exp_circ934 | Primer1 | 5‘- GCAAAGGCAGGCTGCAACTG -3’ | 476 bp |
|  |  | Primer2 | 5‘- GCCCATTCGACCAAAGTGCG-3’ |  |
| 14 | ciri_circ1165,exp_circ935 | Primer1 | 5‘- GCAACCGGTGCAGTCCAAATG -3’ | 454 bp |
|  |  | Primer2 | 5‘- CCTCTCTGCCTCACCTGCTG-3’ |  |
| 15 | ciri_circ1477,exp_circ1266 | Primer1 | 5‘- GCGACAGCGTCGTGTAGATCC -3’ | 321 bp |
|  |  | Primer2 | 5‘- CTGCCCAGGAATGTGGCTCTC-3’ |  |
| 16 | ciri_circ631, exp_circ515 | Primer1 | 5‘- GGGACAAATTCTGCTGCATTTGG -3’ | 585 bp |
|  |  | Primer2 | 5‘- GGCATAGACAGCTTCCTGATG-3’ |  |
| 17 | ciri_circ2462,exp_circ2115 | Primer1 | 5‘- CTGAATGCATCCAGCTGAAAGTTGG -3’ | 336 bp |
|  |  | Primer2 | 5‘- CTCGATTACTGCACAGCGTTGC-3’ |  |
| 18 | ciri_circ2145,exp_circ1854 | Primer1 | 5‘- CTGTCTTGCGAGGACGTGC-3’ | 492 bp |
|  |  | Primer2 | 5‘- CGATGATGCTTCAGAAACCGCAG-3’ |  |
| 19 | ciri_circ1105,exp_circ889 | Primer1 | 5‘- CTGAAAATGTTCCTCAGACAAAATCC -3’ | 336 bp |
|  |  | Primer2 | 5‘- CTGCATCTGCTATGTTGAGTTCACG-3’ |  |
| 20 | ciri_circ1922,exp_circ1629 | Primer1 | 5‘- GCTAGACCAACGGCAAATCCAG -3’ | 233 bp |
|  |  | Primer2 | 5‘- CGATTCGATCACCTTATGGCG-3’ |  |
| 21 | ciri_circ2091,exp_circ1804 | Primer1 | 5‘- GAGCTGGTCAAGCAGCCAC -3’ | 320 bp |
|  |  | Primer2 | 5‘- GATCAACGGCCTCGAGCACGAC -3’ |  |
| 22 | ciri_circ2162,exp_circ1863 | Primer1 | 5‘- CCTTGATACAGATGCAAGATTCATCCC -3’ | 479 bp |
|  |  | Primer2 | 5‘- GCATTTTACTTGGCTTTGCAGCTC -3’ |  |
| 23 | ciri_circ741, exp_circ620 | Primer1 | 5‘- CTCGATTTCCATTACCGCTAGAC -3’ | 327 bp |
|  |  | Primer2 | 5‘- CAAGCTCGTTCTTGAGAAAGTGAC-3’ |  |
| 24 | ciri_circ1309,exp_circ1108 | Primer1 | 5‘- GCAAGAACTCCACTTTCGAAGAATCG -3’ | 538 bp |
|  |  | Primer2 | 5‘- GGAGCTTGCACAAAGCATCG-3’ |  |
| 25 | ciri_circ948, exp_circ790 | Primer1 | 5‘- CTCAGATATCAGCGCATTAACAGC -3’ | 479 bp |
|  |  | Primer2 | 5‘-CCCTCAAGGCACACTAACTTAGC-3’ |  |
| 26 | ciri_circ1977,exp_circ1682 | Primer1 | 5‘- CTCACATCTGTGACTGTTGACTC -3’ | 354 bp |
|  |  | Primer2 | 5‘- GAGCAAGAGAAGAACATAGAGGCAG-3’ |  |
| 27 | ciri_circ2437,exp_circ2095 | Primer1 | 5‘- CTGGCATCACGGAAACCTGTC-3’ | 242 bp |
|  |  | Primer2 | 5‘- TTGGCACGTGGTGGTCAAG-3’ |  |

**Table S6: Alternative splicing of circRNAs**

|  | Number of parental genes | Parental genes with >1 circRNA | Parental genes of >1 circRNAs | Parental genes of 1 circRNAs |
| --- | --- | --- | --- | --- |
| MH63 | 2860 | 732 | 33.6581‬% | 66.3419% |
| ZS97 | 2511 | 606 | 31.8219% | 68.1781% |
| SY63 | 2621 | 695 | 33.696% | 66.3040% |

**Table S7: Genomic distribution of circRNAs**

|  |  | **MH63** |  |  | **ZS97** |  |  | **SY63** |  |
| --- | --- | --- | --- | --- | --- | --- | --- | --- | --- |
| **Chromosome** | **CircRNAs** | **bp/circRNA** | **Genes/circRNA** | **CircRNAs** | **bp/circRNA** | **Genes/circRNA** | **CircRNAs** | **bp/circRNA** | **Genes/circRNA** |
| 1 | 786 | 56631.4606 | 9.13867684 | 625 | 71171.7008 | 11.4864 | 722 | 61651.4238 | 9.94875346 |
| 2 | 608 | 60314.6053 | 9.73848684 | 571 | 65193.3503 | 10.8704028 | 541 | 67784.2514 | 10.9445471 |
| 3 | 643 | 61199.8289 | 9.7807154 | 555 | 70769.8018 | 11.2666667 | 583 | 67498.2676 | 10.787307 |
| 4 | 494 | 73213.0587 | 11.2935223 | 360 | 100975.158 | 15.5416667 | 455 | 79488.4637 | 12.2615385 |
| 5 | 417 | 74056.458 | 11.4028777 | 313 | 97543.9808 | 15.4440895 | 383 | 80630.6606 | 12.4151436 |
| 6 | 451 | 70183.6031 | 10.9844789 | 347 | 92511.4669 | 13.6541787 | 387 | 81790.1938 | 12.8010336 |
| 7 | 360 | 83030.6028 | 13.4277778 | 279 | 107750.713 | 17.7311828 | 324 | 92256.2253 | 14.9197531 |
| 8 | 332 | 89751.6175 | 13.7439759 | 296 | 102079.172 | 16.1148649 | 341 | 87382.8065 | 13.3812317 |
| 9 | 256 | 95048.3125 | 15.0976563 | 238 | 94757.5504 | 15.5630252 | 255 | 95421.051 | 15.1568627 |
| 10 | 271 | 92720.3469 | 14.1808118 | 241 | 106304.216 | 16.9958506 | 254 | 98926.0394 | 15.1299213 |
| 11 | 238 | 138164.58 | 19.3193277 | 212 | 150043.505 | 21.3537736 | 210 | 156586.524 | 21.8952381 |
| 12 | 266 | 98332.1654 | 14.056391 | 233 | 116304.322 | 17.3690987 | 246 | 106326.65 | 15.199187 |

**Table S8: Genomic distribution of lncRNAs**

|  |  | **MH63** |  |  | **ZS97** |  |  | **SY63** |  |
| --- | --- | --- | --- | --- | --- | --- | --- | --- | --- |
| **Chromosome** | **lncRNAs** | **bp/lncRNAs** | **Genes/lncRNAs** | **lncRNAs** | **bp/lncRNAs** | **Genes/lncRNAs** | **lncRNAs** | **bp/lncRNAs** | **Genes/lncRNAs** |
| 1 | 836 | 53244.4115 | 8.59210526 | 916 | 48561.4771 | 7.83733624 | 934 | 47657.7388 | 7.69057816 |
| 2 | 730 | 50234.6301 | 8.1109589 | 680 | 54743.2397 | 9.12794118 | 839 | 43708.3194 | 7.05721097 |
| 3 | 655 | 60078.6107 | 9.60152672 | 674 | 58274.8368 | 9.27744807 | 754 | 52190.305 | 8.34084881 |
| 4 | 860 | 42054.943 | 6.4872093 | 780 | 46603.9192 | 7.17307692 | 919 | 39355.0065 | 6.07072905 |
| 5 | 597 | 51727.8777 | 7.96482412 | 620 | 49243.9774 | 7.79677419 | 655 | 47147.3939 | 7.25954198 |
| 6 | 656 | 48251.2271 | 7.55182927 | 757 | 42406.181 | 6.25891678 | 784 | 40373.4758 | 6.31887755 |
| 7 | 577 | 51804.1889 | 8.37781629 | 661 | 45480.2557 | 7.48411498 | 702 | 42579.7963 | 6.88603989 |
| 8 | 664 | 44875.8087 | 6.87198795 | 654 | 46200.9709 | 7.29357798 | 796 | 37434.0917 | 5.73241206 |
| 9 | 521 | 46703.2015 | 7.4184261 | 481 | 46886.2723 | 7.7006237 | 564 | 43142.4965 | 6.85283688 |
| 10 | 441 | 56977.8095 | 8.71428571 | 446 | 57442.4126 | 9.1838565 | 503 | 49954.6998 | 7.64015905 |
| 11 | 827 | 39761.9952 | 5.5598549 | 746 | 42639.7091 | 6.06836461 | 920 | 35742.5761 | 4.99782609 |
| 12 | 883 | 29622.1472 | 4.23442809 | 812 | 33373.0382 | 4.98399015 | 947 | 27620.2281 | 3.94825766 |

**Table S9: Conservation of lncRNAs and circRNAs in MH63 with other species**

| Proportion of conserved ncRNAs | circRNAs | | lncRNAs | |
| --- | --- | --- | --- | --- |
|  | Genome (%) | Sequence (%) | Genome (%) | Sequence (%) |
| *ZS97* | 92 | 38.69 | 96 | 47 |
| *SY63* | - | 50.82 | - | 58 |
| *O.rufipogon* | 87 | - | 93 | - |
| *O.s.japonica* | 86 | - | 94 | - |
| *O.barthii* | 80 | - | 88 | - |
| *O.glaberrima* | 73 | - | 82 | - |
| *S.bicolor* | 18 | - | 12 | - |
| *S. italica* | 18 | - | 15 | - |
| *B.distachyon* | 15 | - | 14 | - |
| *Z.mays* | 15 | - | 11 | - |
| *P.trichocarpa* | 2 | - | 1 | - |
| *A.thaliana* | 1 | - | 2 | - |

**Table S10: The proportion of Number/length attributable to different TE superfamilies of TE-lncRNAs/circRNAs**

| TE class | Number of TE-lncRNAs | %total length of TE-lncRNAs | Number of TE-circRNA | %total length of TE-circRNA | %total length of TE in the genome |
| --- | --- | --- | --- | --- | --- |
| DNA | 248 | 2.94 | 54 | 4.00 | 2.65 |
| DNA/CMC | 281 | 4.66 | 36 | 3.62 | 7.71 |
| DNA/hAT | 245 | 2.81 | 41 | 2.93 | 2.43 |
| DNA/MULE | 542 | 9.47 | 155 | 12.10 | 8.52 |
| DNA/PIF | 276 | 4.10 | 71 | 4.28 | 3.79 |
| DNA/TcMar | 239 | 3.42 | 103 | 4.72 | 2.67 |
| LINE/L1 | 144 | 2.30 | 48 | 2.83 | 2.31 |
| LTR/Copia | 177 | 6.30 | 30 | 5.36 | 6.67 |
| LTR/Gypsy | 734 | 42.07 | 155 | 33.81 | 46.78 |
| LTR/other | 103 | 2.10 | 12 | 1.41 | 1.19 |
| SINE | 68 | 0.156 | 8 | 0.11 | 0.52 |
| MITE | 661 | 7.50 | 211 | 11.23 | 6.77 |
| other | 26 | 1.16 | 21 | 0.56 | 0.98 |
| RC/Helitron | 643 | 11.02 | 161 | 13.04 | 7.02 |

## 
